# Comprehensive Genomic Survey, Characterization and Expression Analysis of the HECT Gene Family in *Brassica rapa* L. and *Brassica oleracea* L.

**DOI:** 10.3390/genes10050400

**Published:** 2019-05-27

**Authors:** Intikhab Alam, Dong-Li Cui, Khadija Batool, Yan-Qing Yang, Yun-Hai Lu

**Affiliations:** 1 Key Laboratory of Ministry of Education for Genetics, Breeding and Multiple Utilization of Crops, College of Crop Science, Fujian Agriculture and Forestry University, Fuzhou 350002, China; intikhabalam2013@gmail.com (I.A.); donglicui2018@163.com (D.-L.C.); Yanqingyang2018@163.com (Y.-Q.Y.); 2 State Key Laboratory of Ecological Pest Control for Fujian and Taiwan Crops, College of Life Sciences, Key Lab of Biopesticides and Chemical Biology, Fujian Agriculture and Forestry University, Fuzhou 350002, China; khadijabatoolali@gmail.com

**Keywords:** *Brassica*, HECT genes, gene duplication, evolution, gene expression, abiotic stress

## Abstract

The HECT-domain protein family is one of the most important classes of E3 ligases. While the roles of this family in human diseases have been intensively studied, the information for plant HECTs is limited. In the present study, we performed the identification of HECT genes in *Brassica rapa* and *Brassica oleracea*, followed by analysis of phylogeny, gene structure, additional domains, putative cis-regulatory elements, chromosomal location, synteny, and expression. Ten and 13 HECT genes were respectively identified in *B. rapa* and *B. oleracea* and then resolved into seven groups along with their *Arabidopsis* orthologs by phylogenetic analysis. This classification is well supported by analyses of gene structure, motif composition within the HECT domain and additional protein domains. Ka/Ks ratio analysis showed that these HECT genes primarily underwent purifying selection with varied selection pressures resulting in different rates of evolution. RNA-Seq data analysis showed that the overwhelming majority of them were constitutively expressed in all tested tissues. qRT-PCR based expression analysis of the 10 *B. rapa* HECT genes under salt and drought stress conditions showed that all of them were responsive to the two stress treatments, which was consistent with their promoter sequence analysis revealing the presence of an important number of phytohormone-responsive and stress-related cis-regulatory elements. Our study provides useful information and lays the foundation for further functional determination of each HECT gene across the *Brassica* species.

## 1. Background

The posttranslational modification of target proteins by the ubiquitin-proteasome pathway plays an important role in the regulation of plant growth, development and in response to a variety of environmental stresses [1,2,3]. In this pathway, ubiquitin (Ub) is first activated by an ubiquitin-activating enzyme (E1) in an ATP-dependent reaction, where a thioester linkage is formed between the C-terminus of Ub and the active site cysteine (Cys) of E1. Ub is then transthiolated to the active site of Ub-conjugating enzymes (E2), generating an E2~Ub thioester. Specificity of Ub modification is achieved largely through Ub protein ligases (E3), which interacts with both E2~Ub and the substrate to which Ub is to be transferred [4,5,6]. According to the sequences involved in their E2-conjugating domains, E3s Ub ligases can be classified into three main families: (1) HECT-E3s (homologous to E6-AP C Terminus), RING finger-E3s (Really interesting new gene), and U box E3s [7,8,9].

The HECT-domain protein family is one of the most important classes of E3 ligases, defined by the C-terminal region of HECT domain, about 350 amino acids long, which is important for their Ub-ligase activity [8]. The HECT domain is present throughout eukaryotes and is exclusive to HECT E3 ligases [10]. A key feature of the HECT ligases is a conserved Cys residue that forms an intermediate thioester bond with the ubiquitin C terminus before catalysing substrate ubiquitylation [8]. The HECT domain was first defined in human papillomavirus (HPV) E6-associated protein (E6AP) [11]. HECT domain-containing Ub-protein ligases (UPLs) play an important role in human disease or disease-related processes including cancer, cardiovascular and neurological disorders, viral infections, and immune responses [12,13]. Five and 28 HECT genes have been identified in the genomes of *Saccharomyces cerevisiae* and human, respectively [13]. In *Arabidopsis*, seven HECT genes (*UPL1*–*UPL7*) were identified [14], of which *UPL3* was shown to be involved in trichome development [14] and genome endoreduplication [15], *UPL5* in leaf senescence [16], and *UPL1*, *UPL2* and *UPL5* in plant immunity [17]. These seven *Arabidopsis thaliana* HECT genes were classified into four subfamilies according to both HECT domain sequence similarity and the presence of additional domains [14]. Marín [18] analyzed 413 HECT genes derived from various representative viridiplantae species and divided them into six main subfamilies, which might have arisen before the split, separating green algae from the rest of the plants. Meng et al. [19] analyzed 365 plant HECT genes, including 19 HECT genes from the soybean genome (2*n* = 4*x* = 40), and divided them into seven phylogenetic groups. Xu et al. [20] identified 13 HECT genes from the apple genome (2*n* = 2*x* = 34) and divided them into four phylogenetic groups, consistent with those of *A. thaliana*.

Brassicaceae is a large family of plants, composed of 338 genera and 3709 species [21]. The *Brassica* genus includes very important plants, used worldwide due to economically valuable products such as oilseeds, condiments, and other vegetables. Six *Brassica* species (three diploids and three tetraploids) are widely cultivated. The three diploid species *Brassica rapa* (AA, 2n = 20), *Brassica nigra* (BB, 2n = 16), and *Brassica oleracea* (CC, 20 = 18) formed the amphidiploid species *Brassica juncea* (AABB, 2n = 36), *Brassica napus* (AACC, 2n = 38), and *Brassica carinata* (BBCC, 2n = 34) probably by independent natural hybridizations as previously described in the U’s triangle theory [22]. *Brassica* species are rich in morphotypes such as leafy head formation, enlargement of organs, extensive axillary branching, etc., which can be explained by the mixing and evolution of their diverse genomic compositions as well as by the artificial selection during domestication [21,23]. The earlier studies showed that *Brassica*/*Arabidopsis* arose about 20 million years ago (Mya). This was followed by a whole genome triplication (WGT) at approximately 15.9 Mya, divergence of *B. nigra* from other *Brassica* species at approximately 8 Mya, and divergence of *B. oleracea* from *B. rapa* at approximately 4.6 Mya [24,25,26]. The WGT event brought an increase in genomic materials in *Brassica* species, which provides an excellent model to investigate the expansion and evolution of gene families in plants [27]. On the other hand, the occurrence of tandem duplications (TDs) is another important mechanism that leads to the expansion of gene families [28]. The availability of whole genome sequence data for more and more economically important plants makes it possible to realize the genome-wide identification and functional characterization of many important gene families in these plants [29,30,31]. The whole genome sequencing of *B. rapa* in 2011 [32] and *B. oleracea* in 2014 [25] has allowed scientists to investigate and compare the genome conservation, evolution, and arrangements among these Brassicaceae species. As *B. rapa* and *B. oleracea* are closely related to the model plant *A. thaliana*, the homologous cloning approach can easily be used in *Brassica* species based on the sequences (DNA or protein) of well-known and well-characterized *A. thaliana* genes.

Abiotic stresses, especially salt, and drought stresses, affect virtually every aspect of plant physiology and metabolism and cause severe crop yield losses throughout the world [33]. Among the major food crops, *Brassica* crops are mostly affected by drought and salinity, due to the fact that they are mainly grown in arid and semiarid areas [34]. Several drought or salt-tolerant genes have been isolated in the model plant *A. thaliana* as well as in *Brassica* crops, some of which showed great potential for genetic improvement of plant tolerance [34]. Molecular breeding approaches, such as gene transformation, can be integrated in the development of drought- and salt-tolerant *Brassica* crops. Several HECT genes were found to be quite sensitive to abiotic stresses, including salt and drought stresses, suggesting that these HECT family genes might be vital for the response and adaptation to abiotic stresses in plants [20]. In the present paper, a comprehensive analysis of the HECT genes in both *B. rapa* and *B. oleracea* is performed, including the genome-wide identification, gene ontology analysis, chromosome location, phylogenetic classification, syntenic relationships between *B. rapa*, *B. oleracea* and *A. thaliana*, and expression analysis in different tissues and under salt and drought stresses of *B. rapa* HECTs. Our results lay a foundation for further functional characterization of each HECT genes in *Brassica* species and provide useful information for better understanding the function and evolution of this gene family in higher plants, and may help to select the appropriate gene targets for further genetic engineering and genetic improvement of *Brassica* crops.

## 2. Materials and Methods

### 2.1. Identification of HECT Proteins in B. rapa and B. oleracea

To find out the complete set of HECT family proteins in *B. rapa* and *B. oleracea*, two different approaches were used. Initially, all previously identified *Arabidopsis* HECT proteins [14] were downloaded from the *Arabidopsis* database TAIR (http://www. arabidopsis.org) and then used as query sequences for BLASTp searches against the *B. rapa* proteome database at BRAD (http://brassicadb.org/brad/) and *B. oleracea* at Bolbase (http://ocri-genomics.org/bolbase). Second, all identified *Arabidopsis* HECT domains were used as query sequences for BLASTp searches against the same *B. rapa* and *B. oleracea* proteome databases. In some cases, DNA sequences were used as query sequences for BLASTn searches against the *B. rapa* or *B. oleracea* reference genome database for verification or identification of full-length HECT genes. The irredundant sequences retrieved from the two strategies were then analyzed online by SMART (http://smart.embl-heidelberg.de) and Interpro (http://www.ebi.ac.uk/interpro/) to confirm the existence of HECT domains. For some HECT genes with partial-length sequences compared to their *A. thaliana* orthologs or *Brassica* paralogs, we re-annotated their genomic DNA sequences from the corresponding scaffolds by FGENESH v2.6 [35] with the gene-finding parameters of *B. rapa*. The protein sequences of all HECT genes analyzed in this study are provided in Figure S6. For each identified HECT protein, their size, amino acid properties, charge, molecular weight (kDa), aliphatic and instability indice, pI, and grand average of hydropathy (GRAVY) were determined using the online available ProtParam tool (http://web.expasy.org/prot param/), and their associated Gene Ontology (GO) and subcellular location were predicted by using the online CELLO2GO software (http://cello.life.nctu.edu.tw/cello2go/alignment.php).

### 2.2. Multiple Sequence Alignment and Phylogenetic Analysis

A multiple HECT protein sequence alignment was obtained by using the online Clustal Omega program (http://www.ebi.ac.uk /Tools/msa/clustalo/) with default parameters. The best maximum likelihood (ML) tree was predicted by using the online IQ-TREE v1.6.9 software at http://iqtree.cibiv.univie.ac.at/ [36], in which ModelFinder [37] was used to determine the best model of amino acid substitution based on Akaike Information Criterion (AIC), Corrected Akaike Information Criterion (AICc) and Bayesian information criterion (BIC) scores. All three criteria suggested that the General Time Reversible model with empirical frequency, invariable site and under γ rate (GTR + F + I + G4) model was the best-fit model. The branch support values (%) were estimated by both SH-like approximate likelihood ratio test (SH-aLRT) [38] and ultrafast bootstrap (UFBoot) [39] with 1000 replicates each. Three perturbation strengths (0.2, 0.5 and 0.8) were tested to compare the stability of tree topology and determine the optimal tree. The resulting tree was visualized in FigTree v1.4.3 (http://tree.bio.ed.ac.uk/software/figtree/) using the midpoint rooting option.

### 2.3. Gene Exon/Intron Structure Determination and Motif Analysis

The exon/intron structure of each HECT gene was generated by using the online Gene Structure Display Server 2.0 (http://gsds.cbi.pku.edu.cn/). The conserved motifs of HECT domains were identified and analyzed using the MEME tool (v4.11.4; http://meme-suite.org/tools/meme) with the option of 15 motifs. Sequence logos were created based on the alignments of conserved motifs by using the online WebLogo tool (http://weblogo.berkeley.edu/logo.cgi).

### 2.4. Promoter Region Analysis

The promoter sequences (2-kb genomic DNA sequences upstream of the transcription initiation site) of each *B. rapa* and *B. oleracea* HECT genes were retrieved from BRAD (http://brassicadb.org/brad/) and Bolbase (http://ocri-genomics.org/bolbase), respectively. The generic files were then analyzed at the Plant-CARE database (http://bioinfo rmatics.psb.ugent.be/webtools/plantcare/html/) to identify the cis-acting regulatory elements.

### 2.5. Chromosome Location of HECT Genes in B. rapa and B. oleracea

The chromosome location data of each *B. rapa* and *B. oleracea* HECT genes were downloaded from the BRAD and Bolbase database, respectively. Those genes that were assigned to unassembled genomic scaffolds (absence of chromosomal position) were removed from the dataset while the remaining genes were mapped into the specific chromosomes of *B. rapa* and *B. oleracea* by using Circos software [40]. Tandem repeated and segmentally duplicated genes were indicated by differently colored lines.

### 2.6. Syntenic Relationships between B. rapa, B. oleracea, and A. thaliana HECT Genes

For each predicted *B. rapa*, and *B. oleracea* HECT gene, we used the Search Syntenic Gene function of the BRAD database to find its corresponding *A. thaliana* syntenic gene (if it existed). On the other hand, for each known *A. thaliana* HECT gene, we used the same function to find its corresponding *B. rapa* and *B. oleracea* syntenic gene(s) (if existed). In each query, the information about the corresponding syntenic gene name(s) in *A. thaliana*, *B. rapa* and *B. oleracea*, their localization on tPCK (Translocation Proto-Calepineae Karyotype) chromosomes and ancestral chromosome blocks, LF (least fractioned), MF1 (medium fractionated) and MF2 (most fractionated) subgenomes [41,42,43] as well as the eventual tandem repeats in the three species were recorded and summarized in a table.

### 2.7. Calculation of Ka and Ks

The full-length protein sequence alignments were performed by Clustal Omega (http://www.ebi.ac.uk/Tools/msa/clustalo/). We estimated the synonymous substitution rate (Ks), non-synonymous substitution rate (Ka), and evolutionary constriction (Ka/Ks) between the orthologous *A. thaliana* and *B. rapa* or *B. oleracea* HECT gene pairs using PAL2NAL and codeml in the PAML package (http://www.bork.embl.de/pal2nal/index.cgi?example=Yes#RunP2N) [44,45]. The divergence time was calculated through formula T = Ks/2R, where T is divergence time, Ks is to the synonymous substitutions per site, R is intended for the divergence rate of nuclear genes from plants, and the *R*-value is considered as 1.5 × 10 ^−8^ to 9 × 10 ^−9^ synonymous substitutions per site per year in the case of dicotyledonous plants [46].

### 2.8. Expression Analysis of HECT Genes in B. rapa and B. oleracea

The RNA-Seq expression data across six tissues (callus, root, stem, leaf, flower, and silique) of the *B. rapa* accession Chiifu-401–42 and six tissues of *B. oleracea* were retrieved from the Gene Expression Omnibus (GEO) database of NCBI (http://www.ncbi.nlm.nih.gov/geo/) using the accession number GSE43245 and GSE42891, respectively [26,47]. The expression values (numbers of fragments per kilobase of transcript per million mapped reads, FPKM) of identified *B. rapa* and *B. oleracea* HECT genes were extracted from the dataset and submitted to clustering analysis using Cluster software v3.0 (http://bonsai.hgc.jp/~mdehoon/software/cluster/) using the complete linkage clustering method. The clustering tree together with the gene expression heatmap was generated by using the Java Tree view software (Version 1.1.6r4, http://jtreeview.sourceforge.net/).

### 2.9. Preparation of Plant Materials and qRT-PCR Analyses

For plant samples preparation, *B. rapa* accession Chiifu-401–42 seeds were sterilized and sown in a Petri dish with moisture-absorbent filter papers and incubated at 25 °C. The germinated seedlings were then transferred into plastic pots containing growth medium with vermiculite and peat 3:1 grown in a greenhouse at 22 °C with a photoperiod of 16/8 h for light/dark. Seedlings (21 days old) were used for different abiotic treatments. For salinity and PEG treatments, the plants were irrigated with 200 mM NaCl and 20% (*w*/*v*) polyethylene glycol (PEG 6000), respectively. Then, the leaves from control and stressed plants were harvested after 0, 1, 3, and 24 h of treatments and immediately immersed in liquid nitrogen, and stored at −80 °C until RNA extraction use. For each treatment, three biological replicates were prepared to decrease the error rate.

Total RNA was isolated from about 100 mg of frozen leaves of each sample of *B. rapa* using a Plant RNA Extraction Kit according to the manufacturer’s instructions (OMEGA, China). The quality of RNA was checked by agarose gel electrophoresis and quantified by using the NanoDrop 2000 Spectrophotometer (Thermo Fisher Scientific, Inc., Waltham, MA, USA). First-strand cDNA was synthesized using 1 μg of total RNA per sample with the cDNA Synthesis Kit from TaKaRa Bio Inc. (Dalian, China). The reverse transcription products were diluted 20-fold and stored at −20 °C prior to analysis. Gene-specific primers for the selected *B. rapa* HECT genes were designed using Primer3Plus software (http://www.primer3plus.com/). The *B. rapa Actin*-2 gene (GenBank accession number XM_018658258) was used as an internal reference gene. The primers used for qRT-PCR and their expected amplification product size are summarized in Table S2. The qRT-PCR analysis was performed on an ABI 7500 Fast Real-time PCR amplification system (Applied Biosystems, Foster, CA, USA). The analysis was carried out in a total volume of 20 µL containing 2 µL template of cDNA, 0.8 µL of the forward and reverse primers (10 μM), 10 µL of SYBR Green PCR Master (ROX) (Roche, Shanghai, China), and 6.4 µL of sterile distilled water. The PCR amplification parameters were as follows: 95 °C for 1 min, followed by 40 cycles of 95 °C for 15 s, and 60 °C for 70 s. For each sample, three replicates were run to compute the average Ct values. The data were analyzed by using the 2^−ΔΔCt^ method [48]. Relative gene expression levels were normalized against the expression of the housekeeping gene *BrActin*-2. The significance of differences with *p* < 0.05 among relative expression levels of samples at different time-points of treatment were statistically analyzed by using one-way ANOVA and Tukey HSD test in IBM SPSS software v22.0.

## 3. Results

### 3.1. Genome-Wide Identification of HECT Genes in Brassica rapa and Brassica oleracea

To identify each member of the HECT family in the *B. rapa* and *B. oleracea* genomes, we performed BLASTP and tBLASTn searches using *Arabidopsis* HECT protein sequences as queries against BRAD and Bolbase databases, respectively. The retrieved redundant sequences were removed, and the non-redundant sequences were then analyzed for the presence or absence of the HECT domain by using the Pfam and SMART databases. During the analysis, we found that some putative HECT genes (e.g., *Bra000779*, *Bra005748*, *Bol012097*, *Bol003946*, *Bol016068*, *Bol000411* and *Bol000412*) have only partial-length sequences compared to their *A. thaliana* orthologs and/or *Brassica* paralogs. We then identified their locations on corresponding genomic scaffolds by tBLASTn and re-annotated their genomic sequences by FGENESH 2.6 [35]. We then obtained the full-length sequences for *Bra000779*, *Bra005748*, *Bol012097*, *Bol003946* and *Bol016068* (revised as *Bol16067/8*), and revised sequences for *Bol000411* and *Bol000412*, both of which lack the four first exons (compared to their paralog *Bol003376*) due to their locations on the two extremities of Scaffold000577. Finally, we identified 10 HECT genes in *B. rapa* and 13 in the *B. oleracea* genome, which are summarized in Table S1. Analysis of their protein sequences indicated that these HECT proteins have lengths ranging from 883 to 3784 aa in *B. rapa*, and from 504 to 3546 aa in *B. oleracea*, with computed molecular masses ranged from 101 to 417 kDa in *B. rapa* and from 58 to 390 kDa in *B. oleracea*, and isoelectric points (pIs) ranging from 4.84 to 7.86 in *B. rapa* and from 4.87 to 8.35 in *B. oleracea*. The computed physico-chemical properties by ProtParam tool [49], predicted that nearly all these *Brassica* HECT proteins were hydrophilic (GRAVY < 0) and unstable in a test tube (Instability index > 40, except *Bol007120* with Instability index < 40), but were thermostable (Aliphatic index = 82.6~96.09). Gene Ontology analysis predicted that all these HECT proteins displayed ubiquitin-protein ligase activity, were mainly localized in the nucleus (19/23), plasma membrane (19/23) and/or cytoplasm (3/23) and are involved in a variety of biological processes in the cell, such as cellular protein modification process, catabolic process, cell differentiation, response to stress, etc. (Table S1).

### 3.2. Phylogenetic Analysis

**Figure 1:**
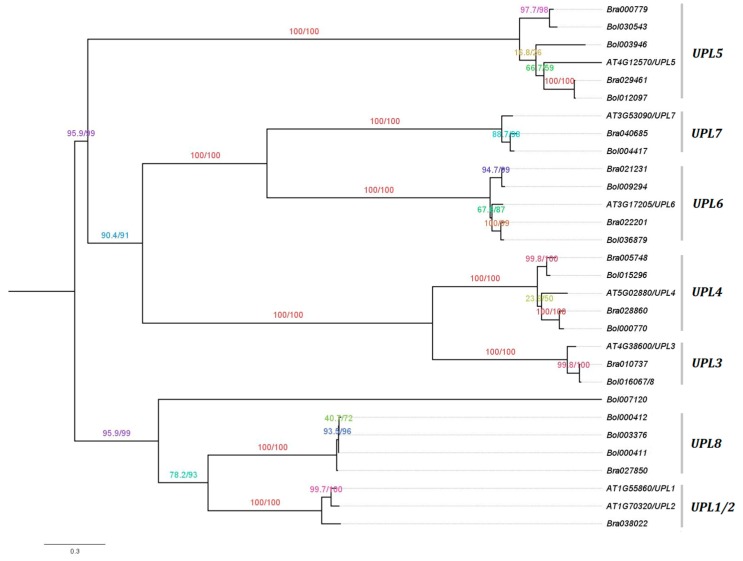
Phylogenetic tree of *Brassica rapa*, *Brassica oleracea* and *Arabidopsis thaliana* HECT genes. The tree was generated by maximum likelihood using the IQ-TREE software [36] with a perturbation strength of 0.8, and visualized in FigTree v1.4.3 (http://tree.bio.ed.ac.uk/software/figtree/) using the midpoint rooting option. The branches that correspond to the seven phylogenetic groups (i.e., *UPL1/2*, *UPL3*, *UPL4*, *UPL5*, *UPL6*, *UPL7* and *UPL8*) defined in a previous study by Marín [18] are labelled. Numbers indicate the SH-aLRT support (%)/ultrafast bootstrap support (%) assessed with 1000 replicates each. The scale bar is indicated at the bottom of the figure. UPL: ubiquitin-protein ligase.

The best maximum likelihood (ML) phylogenetic tree was predicted using the IQ-TREE software [36] for the HECT genes from *B. rapa*, *B. oleracea*, and *A. thaliana*, based on their protein sequences (Figure 1). Both SH-like approximate likelihood ratio test (SH-aLRT) [38] and UFBoot analysis [39] scores indicate that most of the branches (or splits) have high support values ranging from 90 to 100%. The topology of the tree remained stable with three different perturbation strengths of 0.2, 0.5 and 0.8, and revealed that the HECT gene family can be divided into seven phylogenetic groups in accordance with a previous study by Marín [18] (i.e., *UPL1/2*, *UPL3*, *UPL4*, *UPL5*, *UPL6*, *UPL7* and *UPL8*), where the orthologous HECT genes from *B. rapa*, *B. oleracea*, and *A. thaliana* were closely clustered together (Figure 1). We can observe that *B. rapa* contains 1 *UPL1/2*, 1 *UPL3*, 2 *UPL4*, 2 *UPL5*, 2 *UPL6*, 1 *UPL7* and 1 *UPL8* genes, while *B. oleracea* contains 0 *UPL1/2*, 1 *UPL3*, 2 *UPL4*, 3 *UPL5*, 2 *UPL6*, 1 *UPL7* and 3 *UPL8* genes. One *B. oleracea* HECT member, *Bol007120*, was clustered together with the members of *UPL1/2* and *UPL8*, but not into any group (Figure 1).

### 3.3. Gene Structure and Motif Composition Analysis

To characterize the structural diversity of the HECT gene family in *A. thaliana*, *B. rapa* and *B. oleracea*, we analyzed the exon-intron organization of each HECT gene based on the phylogenetic tree in Figure 1. As illustrated in Figure S1, closely related HECT genes generally have similar gene structure with similar exon/intron number, and differed mainly in their respective intron and exon lengths. Members in groups *UPL1/2*, *UPL3*, *UPL4*, *UPL5, UPL6*, and *UPL7* share a common exon/intron number of 15/14, 17/16, 16/15, 3/2, 26/25 and 14/13, respectively. In group *UPL8*, *Bol003376* has an exon/intron number of 16/15, while its ortholog *Bra027850* has a number of 17/16; in addition, the two partial-length genes *Bol000411* and *Bol000412* showed also a difference (by one) in their exon/intron number. Similar gene structures are also observed between the members of groups *UPL1/2* and *UPL8*, and *UPL3* and *UPL4*. We checked intron positions (boundaries) in each HECT gene and found that they were perfectly conserved among the full-length members of each group. One *B. oleracea* HECT gene (*Bol007120*) showed a unique pattern of exon-intron organization compared to all other analyzed HECT genes with 11 exons and 10 introns (Figure S1).

**Figure 2:**
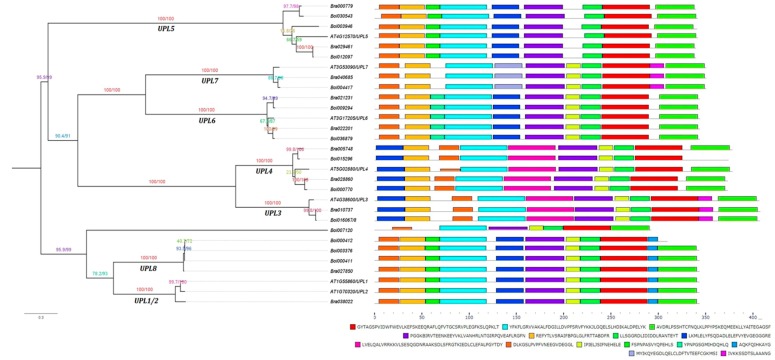
Conserved motif analysis of HECT domains among *B. rapa*, *B. oleracea* and *A. thaliana* HECT proteins. The 15 most conserved motifs were identified by using online the MEME program (http://meme-suite.org/tools/meme). The maximum likelihood phylogenetic tree is provided on the left side of the figure followed by the different motifs, represented by different colors, in the HECT domains of corresponding *A. thaliana*, *B. rapa* and *B. oleracea* HECT proteins on the right side. The black lines showed the non-conserved sequences. The scale bar at the bottom (right side) indicates the lengths of proteins. Sequences of the 15 most conserved motifs are also shown at the bottom.

To characterize the structural diversity of the HECT domains in different HECT genes, we analyzed their first 15 most conserved motifs (Motif 1 to Motif 15) using the online MEME tool (http://meme-suite.org/tools/meme). As illustrated in Figure 2, all the HECT proteins contained the first three most conserved motifs in their HECT domains except one member in group *UPL8* (*Bol000412*) and another member in group *UPL4* (*Bol015296*) in which the third motif was not detected. The members in a same phylogenetic group generally shared a same set of motifs except *Bol000412* in group *UPL8* and *Bol015296* in group *UPL4*. Furthermore, same motif compositions were also observed between the members of groups *UPL1/2* and *UPL8* (except *Bol000412*), and *UPL3* and *UPL4* (except *Bol015296*). *Bol007120* showed a unique motif composition compared to the other members. Sequence logos for these 15 most conserved motifs of HECT domains are shown in Figure S2. Analysis of these 15 motif sequences by SMART showed that they did not correspond to any known domain.

### 3.4. Putative Cis-Regulatory Element Analysis

**Figure 3:**
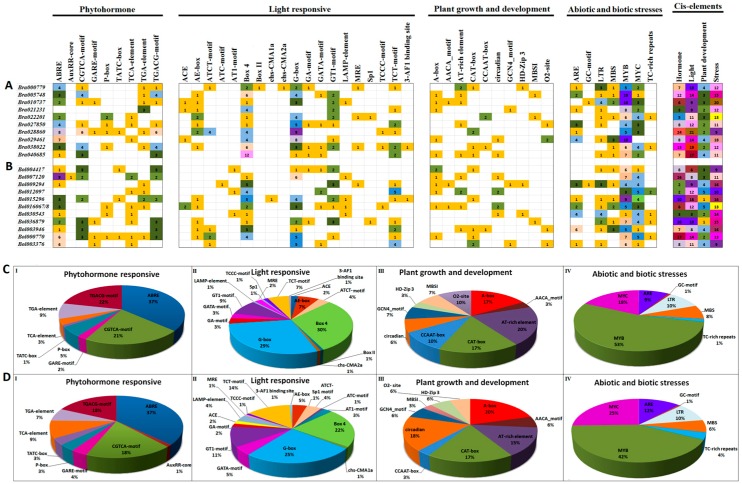
Putative cis-regulatory element analysis of the promoter region (2kb) of 10 *B. rapa* (**A**,**C**) and 11 *B. oleracea* (**B**,**D**) HECT genes. The numbers of different cis-regulatory elements identified in the promoter region of 10 *B. rapa* (**A**,**C**) and 11 *B. oleracea* HECT genes are shown by different colors and numbers in the grid. The sum of the cis-regulatory elements in each category was displayed with a differently colored histogram on the upper right side. The ratio of each identified cis-regulatory element in each category of *B. rapa* (**C**) and *B. oleracea* (**D**) was represented by pie charts.

Analysis of cis-regulatory elements in promoter sequences can give some important clues to understand the function and regulation of the downstream genes. For this reason, the 2-kb genomic DNA sequences upstream of the transcription initiation site of each *B. rapa* and *B. oleracea* HECT genes were retrieved from BRAD and the Bolbase database, respectively. Putative cis-regulatory elements in the 2-kb promoter region of each HECT gene were identified using the PlantCARE database. As illustrated by the Figure 3, the promoter region of each HECT gene contained a unique collection of cis-regulatory elements of different categories. A total of 391 and 374 putative cis-regulatory elements were detected in the promoter regions of 10 *B. rapa* and 11 *B. oleracea* HECT genes, respectively (promoter sequences of *Bol000411* and *Bol000412* are not available). According to their predicted biological processes, these identified cis-regulatory elements can be divided into four categories: phytohormone-responsive (9), light-responsive (19), plant growth and development (10), and stress-responsive (7) (Figure 3A,B). Globally, the growth and development related cis-regulatory elements were detected with a relative low frequency compared to three other categories of cis-regulatory elements. Three phytohormone-related elements (i.e., ABRE, CGTCA and TGACG involving in abscisic acid, methyl jasmonate and auxin signaling, respectively), two light-responsive elements (i.e., G-box and Box 4) and two stress-related elements (i.e., MYB and MYC) were detected with high frequency in the promoter regions of both *B. rapa* and *B. oleracea* HECT genes (Figure 3C,D).

### 3.5. Search for Additional Protein Domains

**Figure 4:**
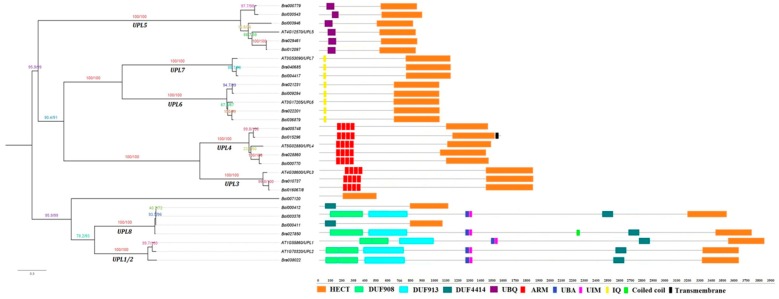
Domain architectures of *B. rapa*, *B. oleracea* and *A. thaliana* HECT proteins and their phylogenetic relationships. Putative additional domains in each HECT protein were predicted by using the online tools of SMART (http://smart.embl-heidelberg.de) and Interpro (http://www.ebi.ac.uk/interpro/) databases. Each domain is represented by a colored box indicated at the bottom. DUF: Domain of unknown function; UBQ: Ubiquitin homologues; ARM: Armadillo repeats; UBA: Ubiquitin-associated domain; UIM: Ubiquitin-interacting motif; IQ: IQ short calmodulin-binding motif. The scale bar at the bottom (right side) indicates the lengths of proteins.

To better classify the identified *B. rapa* and *B. oleracea* HECT proteins, we inspected their full-length protein sequences for additional protein domains, besides the HECT domain, using Smart [50] and InterPro [51]. Ten putative additional domains were identified among the HECT proteins of the three species, namely, the DUF908, DUF913, DUF4414, UBQ, ARM, UBA, UIM and IQ domains (Figure 4). Among them, DUF908, DUF913, DUF4414, UBA, and UIM domains were present together in the members of group *UPL1/2* (except the two partial-length genes *Bol000411* and *Bol000412*) and *UPL8*. UBQ was present in all the six members of group *ULP5*. IQ was present in all the members of group *UPL6* and *UPL7*. ARM-repeats were present in all the members of group *UPL3* and *UPL4*. All the above-mentioned domains have been reported in previous studies [14,18,19,20]. In addition, we detected a Coiled-coil domain in one member of group *UPL8* (*Bra027850*), and a Transmembrane domain in one member of group *UPL4* (*Bol015296*). The member *Bol007120* did not have any additional known domain.

### 3.6. Chromosomal Distribution, Synteny and Evolutionary Analysis of HECT Genes in *Brassica rapa* and *Brassica oleracea*

**Figure 5:**
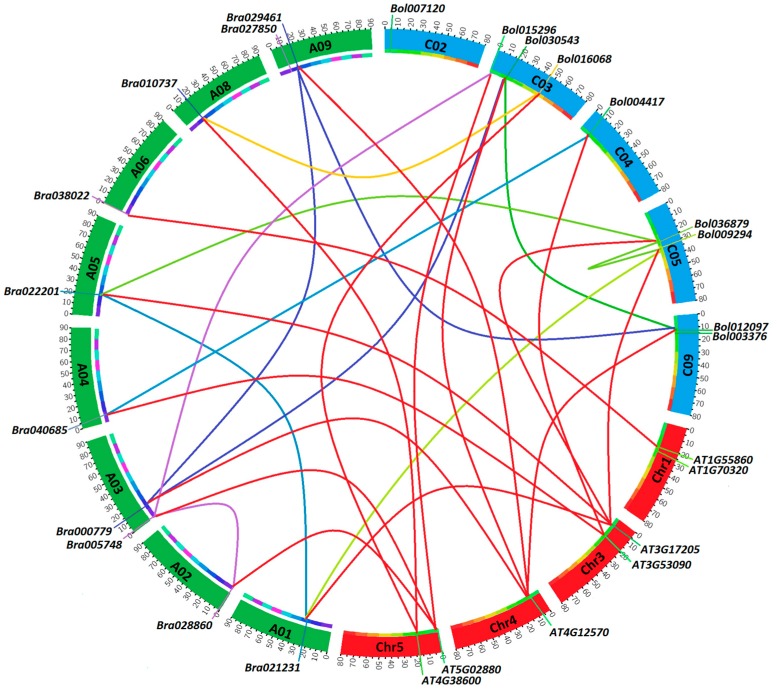
Chromosomal position, synteny, and expansion of HECT genes among *B. rapa*, *B. oleracea*, and *A. thaliana*. Physical locations of each HECT gene are shown across the eight *B. rapa*, five *B. oleracea* and four *A. thaliana* chromosomes. The segmental duplications (within a given species) and syntenic relationships (between two different species) are represented by differently colored lines.

To have a general view of their chromosomal distribution on the genome, we mapped these HECT genes on the corresponding chromosomes according to their physical position data retrieved from the genomic BRAD and Bolbase public databases (Table S1). After curation, 10 HECT genes in *B. rapa* and 8 HECT genes in *B. oleracea* were mapped to respective chromosomes, and compared to the mapping of the seven *A. thaliana* HECT genes (Figure 5). These genes were detected on eight of ten chromosomes in *B. rapa* (i.e., A01–A06 and A08–A09) and five of nine chromosomes in *B. oleracea* (i.e., C02–C05 and C09). Their distributions on the chromosomes were not even but showed a tendency to be localized near the ends of chromosomes in all three species. Five *B. oleracea* HECT genes were not mapped to a specific chromosome as they were actually assigned to isolated scaffolds.

To examine the selection pressures on the HECT genes in *B. rapa* and *B. oleracea*, we calculated the Ka, Ks and Ka/Ks values as well as the divergence times of different orthologous *Arabidopsis*-*Brassica* HECT gene pairs (Table 1). The Ka/Ks values ranged from 0.150 to 0.315 with an average of 0.232 in *B. rapa*, and from 0.158 to 0.363 with an average of 0.246 in *B. oleracea*. All the orthologous gene pairs had Ka/Ks ratios significantly inferior to 1, indicating that they had experienced strong negative or purifying selection in the process of species evolution. The estimated divergence times between orthologous *Arabidopsis* and *Brassica* HECT genes pairs ranged from 8.38~14.06 to 26.43~44.04 Mya, with an average of 14.23~23.72 Mya, which is close to the commonly accepted value of 20 Mya separating *Arabidopsis* and *Brassica* lineages [25]. Furthermore, we generated a Ka/Ks annotated evolutionary tree of all the HECT genes from *A. thaliana*, *B. rapa* and *B. oleracea* using the online Ka/Ks Calculation tool at http://services.cbu.uib.no/tools/kaks with default parameters (Figure S3). The result showed that all branches of the resulting phylogenetic tree have Ka/Ks ratios less than 1.0, indicating that these HECT genes had undergone exclusive purifying selection during their evolution.

### 3.7. Expression Analysis of HECT Genes in B. rapa and B. oleracea

**Figure 6:**
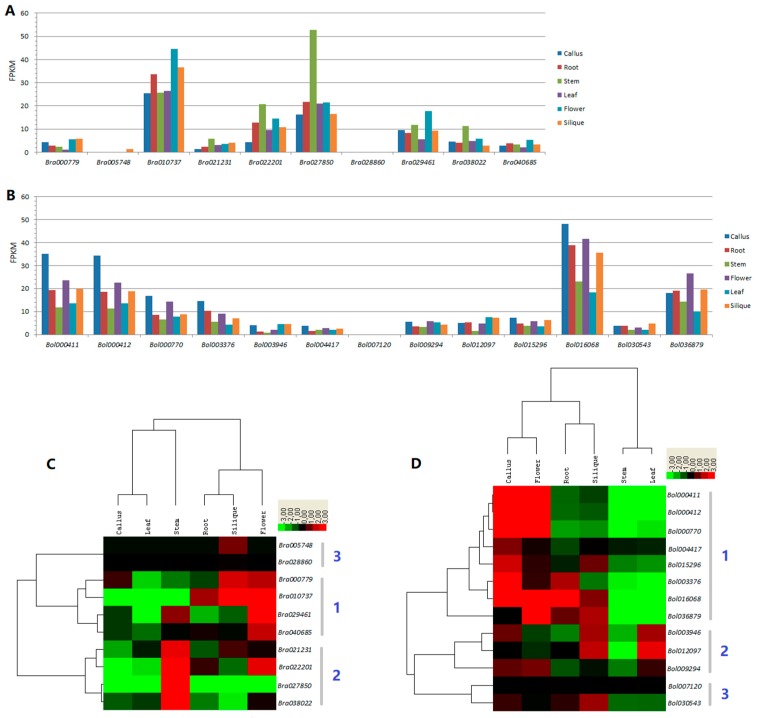
Expression analysis of HECT genes in *B. rapa* (**A**,**C**) and *B. oleracea* (**B**,**D**). The expression profile data of 10 *B. rapa* and 13 *B. oleracea* HECT genes across six different tissues (callus, root, stem, flower, leaf, and silique) were obtained from the Gene Expression Omnibus (GEO) database of NCBI (GSE43245 and GSE42891). Expression levels (numbers of fragments per kilobase of transcript per million mapped reads, FPKM) of each HECT gene in six different tissues were illustrated by histograms in *B. rapa* (**A**) and *B. oleracea* (**B**). Clustered heatmaps were generated for the 10 *B. rapa* (**C**) and 13 *B. oleracea* (**D**) HECT genes based on same sets of FPKM values. Color scale bars representing the relative signal ratios are shown on right top side of each heatmap. The tissue types are indicated on the right side of each histogram figure (**A**,**B**) and top of each heatmap (**C**,**D**). The individual gene names are indicated on the bottom of each histogram figure (**A**,**B**) and right side of each heatmap (**C**,**D**).

Based on their RNA-Seq expression profile data retrieved from the GEO database, histograms displaying the expression levels (numbers of fragments per kilobase of transcript per million mapped reads, FPKM) of the 10 *B. rapa* and 13 *B. oleracea* HECT genes across six different tissues (callus, root, stem, leaf, flower and silique) were generated, as shown in Figure 6A,B. In *B. rapa*, eight out of 10 HECT genes were constitutively expressed in all the six tested tissues, while one (*Bra005748*) was specifically expressed (FPKM = 1.42) in silique, and another one (*Bra028860*) was only poorly expressed (FPKM = 0.19) in silique. *Bra010737* (orthologous to *Arabidopsis UPL3*) showed high expression levels in all the six tested tissues with FPKM values varied from 25 to 45. *Bra027850* (*UPL8*, no corresponding ortholog exists in *Arabidopsis*) was the second most highly expressed HECT member in *B. rapa*, with FPKM values ranging from 16 to 22 for callus, root, leaf, flower, and silique, but with an FPKM as high as 53 for the stem. In *B. oleracea*, 12 out of 13 HECT genes were constitutively expressed in all tested tissues, while one (*Bol07120*) was only very poorly expressed (FPKM = 0.10) in the silique, and was not expressed in the other tissues. *Bol016068* (orthologous to *Arabidopsis UPL3*) showed high expression levels in all six tested tissues with FPKM values from 18 to 48. The two tandem repeated members *Bol000411* and *Bol000412* (*UPL8*, absent in *Arabidopsis*) showed a similar expression pattern across the six tested tissues with FPKM values from 11 to 35.

For comparison, the expression profiles of *Arabidopsis* HECT genes in 47 developmental tissues were retrieved from the TAIR database (https://www.arabidopsis.org/) using the Arabidopsis eFP Browser and summarized in Figure S4. Data for *UPL6* were not available in the database. *UPL1* and *UPL2* shared an identical set of expression data. All six *Arabidopsis* HECT genes were constitutively expressed in all tested tissues. *UPL1*/*UPL2* have the highest expression values ranging from 400 to 600, followed by *UPL3* ranging from 200 to 400, *UPL4* ranging from 100 to 300, and *UPL5*/*UPL7* ranging from 50 to 100. Globally, there is a good correlation between *Arabidopsis* and *Brassica* HECT genes regarding to their expression patterns: the orthologous members tend to maintain similar expression patterns (relative expression levels) across *Arabidopsis* and *Brassica* species. For examples, the members of group *UPL3*, i.e., *Bra010737*, *Bol016086* and *UPL3*, were all constitutively expressed at relatively high levels, while those of group *ULP7*, i.e., *Bra040685*, *Bol004417* and *UPL7*, were all constitutively expressed with relatively lower levels compared to the other HECT genes in *B. rapa*, *B. oleracea* and *A. thaliana* (Figure 1 and Figure 6, Figure S4).

Clustered heatmap analysis showed that both the *B. rapa* and *B. oleracea* HECT genes can be divided into three groups (1–3) (Figure 6C,D) based on their RNA-Seq expression profiles. In both species, group 1 contains the HECT genes (four in *B. rapa* and eight in *B. oleracea*) mainly and preferentially expressed in the flower, and group 3 contains members (two in *B. rapa* and two in *B. oleracea*) preferentially expressed in the silique. However, *B. rapa* group 2 contains four members preferentially expressed in the stem, while *B. oleracea* group 2 contains three members preferentially expressed in the leaf, silique, flower and callus.

### 3.8. Expression Analysis of B. rapa HECT Genes under Abiotic Stresses

**Figure 7:**
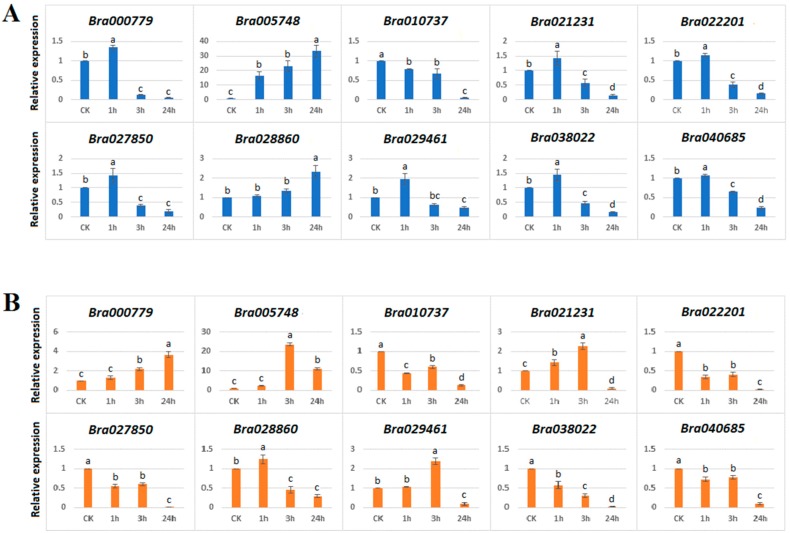
qRT-PCR expression patterns of 10 *B. rapa* HECT genes under salt (**A**) and drought (**B**) treatments. The sampling time-points are on the *x*-axis and the relative expression levels are on the *y*-axis. Statistical significance of differences between control and treated groups was analyzed using Tukey tests. The different letters indicate the statistically significant differences with *p* ≤ 0.05.

To gain information about the response of HECT genes to abiotic stresses, we examined the expression response of 10 *B. rapa* HECT genes to salt (200 mM NaCl) (Figure 7A) and drought (20% (*w*/*v*) PEG_6000_) (Figure 7B) stresses in the leaves of 21-day-old seedlings by using the qRT-PCR technique. The results showed that all the studied 10 *B. rapa* HECT genes were responsive to the two abiotic stress treatments.

In the case of salt treatment, nine *B. rapa* HECT genes were up-regulated, and only one (*Bra010737*) was down-regulated compared to the control (CK) after 1 h, 3 h or 24 h of treatment (Figure 7A). Interestingly, the paralogous genes *Bra00779* and *Bra029461* (orthologous to *AT4G12570*/*UPL5*) showed similar expression patterns in that both were moderately up-regulated under salt treatment at 1 h and then down-regulated at 3 and 24 h; the paralogous genes *Bra005748* and *Bra028860* (orthologous to *AT5G02880*/*UPL4*) also showed similar expression patterns in that both were progressively inducted along with the time under salt treatment, but the former reached a high level of 32-fold of the control at 24 h, while the latter reached only 2.2-fold of the control at 24 h; the paralogous genes *Bra021231* and *Bra022201* (orthologous to *AT3G17205*/*UPL6*) also showed very similar expression patterns in that both were moderately up-regulated at 1 h of treatment but significantly down-regulated at 3 h and 24 h.

In the case of exposure to drought stress, all 10 *B. rapa* HECT genes were responsive to treatment with 5 genes up-regulated and 5 down-regulated compared to the control (CK) after 1 h, 3 h or 24 h of treatment (Figure 7B). The highest induction was recorded for *Bra005748*, which showed >22-fold up-regulation at 3 h and retained up to 9-fold up-regulation at 24 h compared to the control, while its paralog *Bra028860* was only slightly up-regulated (1.2-fold) at 1 h, but significantly down-regulated at 3 and 24 h. Furthermore, the gene *Bra00779* was progressively inducted over time and reached 3-fold at 24 h compared to the control, while its paralog *Bra029461* retained the expression up to 2-fold at 3 h but was down-regulated at 24 h. In another case, the gene *Bra021231* was first up-regulated at 1 h and 3 h up to 2.2-fold and then down-regulated at 24 h while its paralogous gene *Bra022201* was down-regulated in all treatments compared to the control.

For comparison, we retrieved the expression patterns of *Arabidopsis* HECT genes in shoots (16 days after germination) under salt and drought stresses from the TAIR database (https://www.arabidopsis.org/) using the *Arabidopsis* eFP Browser, summarized in Figure S5. *UPL1* and *UPL2* share an identical set of data, while *UPL6* is absent in the database. Salt stress was performed by transferring the plant to a medium containing 150 mM NaCl, while drought stress was performed by exposing the plant to the air stream for 15 min with loss of approximately 10% of fresh weight. The results showed that all tested *Arabidopsis* HECT genes were responsive to both stresses and were more or less up-regulated upon the treatments followed by decrease of expression level. Their expression patterns were somewhat similar between the two stresses. Coincident with its *B. rapa* ortholog *Bra028860*, *A. thaliana UPL4* was the most sensitive member by an up-regulation of expression up to approximatively 2-fold after 3 to 6 h of salt or drought treatments (Figure S5).

## 4. Discussion

In the current study, 10 and 13 HECT genes were identified in the genomes of *B. rapa* and *B. oleracea*, i.e., respectively, 1.4 and 1.8 times the number (7) of genes in *A. thaliana*. Phylogenetic analysis based on protein sequences resolved them into seven groups along with the seven *A. thaliana* HECT genes (Figure 1). This classification is well supported by analyses of gene structure (Figure S1), motif composition of HECT domain (Figure 2) and additional protein domains (Figure 4), and it corresponds well with the previous studies on plant HECT genes [14,18,19]. Compared with the classification of Marín [18], our groups *UPL3* and *UPL4* can be integrated into his subfamily I, group *UPL7* into his subfamily II, group *UPL6* into his subfamily III, groups *UPL1/2* and *UPL8* into his subfamily V, and group *UPL5* into his subfamily VI. As the seven *A. thaliana* and 10 *B. rapa* HECT genes were also included in the phylogenetic analysis of Meng et al. [19] in soybean, we can easily state our groups of *UPL1/2*, *UPL3*, *UPL4*, *UPL5*, *UPL6*, *UPL7* and *UPL8* correspond to their groups I, VI, VII, III, IV, V and II, respectively. Compared with the previous studies in *A. thaliana* [14] and apple [20], our groups *UPL1/2* and *UPL8*, *UPL3* and *UPL4*, and *UPL6* and *UPL7* are merged into three single groups in their analyses.

The *Brassica* ancestor deviated from a common ancestor with *A. thaliana* approximately 20 Mya and underwent a WGT at approximately 15.9 Mya followed by intensive gene loss [24,25,26]. *B. rapa* and *B. oleracea* diverged from a common ancestor at approximately 4.6 Mya, and then experienced parallel evolution [26]. A previous study showed that the common ancestor of all plants contained seven HECT genes, and the HECT gene family has followed a very conservative evolutionary pattern, in which a small number of ancestral HECT genes have been well conserved during evolution in most lineages despite the numerous genome duplications in higher plants [18]. Our analysis showed that, after intensive gene loss following the WGT event, *B. rapa* conserved 1 *UPL1/2*, 1 *UPL3*, 2 *UPL4*, 2 *UPL5*, 2 *UPL6*, 1 *UPL7* and 1 *UPL8* genes, while *B. oleracea* conserved 0 *UPL1/2*, 1 *UPL3*, 2 *UPL4*, 3 *UPL5*, 2 *UPL6*, 1 *UPL7* and 3 *UPL8* genes (Figure 1). The differences in the number of HECT genes between the two species (if not due to incomplete sequencing of the genomes) may be due to natural selection or simply to random differential elimination of partially redundant genes. The tandemly repeated HECT gene pair *Bol000411*-*Bol000412* in *B. oleracea* corresponds to *Bra027850*-*Bra027853* in *B. rapa*, but *Bra027853* is a shortened copy in which the expected HECT domain was absent, and therefore was not detected as a HECT gene (data not shown). *Bol007120* displayed a unique exon/intron structure compared to other HECT members (Figure S1), and no corresponding orthologous copy was found in *B. rapa* and *A. thaliana*, but it was detected in *B. napus* (corresponding to *BnaCnng42680D*). Further verification will be necessary to confirm if this is due to incomplete genome sequencing. On the other hand, the Ka/Ks ratios calculated for the orthologous HECT gene pairs between *A. thaliana* and *Brassica* species (Table 1), as well as for all the branches of the Ka/Ks annotated evolutionary tree of HECT genes from *A. thaliana*, *B. rapa* and *B. oleracea* (Figure S3), were inferior to 1, indicating that these HECT genes have mainly undergone purifying selection during evolution. The significant variation in Ka/Ks ratios observed for different *Brassica* HECT genes, especially the WGT-caused triplicated members (ex. *Bol012097*, *Bol030543*, and *Bol003946*), suggested that they had evolved under different selection pressures resulting in different rates of evolution (Table 1).

RNA-Seq based expression analysis showed that the overwhelming majority of these *Brassica* HECT genes (8 out of 10 in *B. rapa*, and 12 out of 13 in *B. oleracea*) were constitutively expressed in all tested tissues (Figure 6A,B), similar to their *Arabidopsis* orthologs (Figure S4), indicating that all these HECT genes might function as part of the most fundamental, housekeeping, cellular machinery [18], and can be involved in all the stages of plant growth and development. Orthologous HECT genes from *B. rapa*, *B. oleracea*, and *A. thaliana* tend to conserve similar expression patterns, suggesting that their function might also be conserved in the three species. Another observation is that the triplicated HECT genes diverged in expression, with one member being more highly expressed than the others. For example, in group *UPL6*, *Bra022201* is more highly expressed than its duplicated paralog *Bra021231* in *B. rapa*, while the corresponding ortholog *Bol036879* is also more highly expressed than its duplicated paralog *Bol009294* in *B. oleracea* (Figure 6A,B). This is consistent with a previous study on the RING finger protein gene family [30]. On the other hand, our clustered heatmap analysis showed some marked differences between *B. rapa* and *B. oleracea* (Figure 6C,D), suggesting that the function of some HECT members (especially the duplicated pairs) may have diverged between the two species. One *B. olelacea* HECT gene, *Bol007120*, displaying a unique exon/intron composition pattern compared to the other *Brassica* HECT genes (Figure S1), was only very poorly expressed in the silique (Figure 6B), implying that it may correspond to a degenerated HECT member that plays a minor role in plant growth and development.

Our analysis of expression patterns of 10 *B. rapa* HECT genes in response to salt and drought stresses showed that all they were responsive to the treatments of two abiotic stresses with different amplitudes and varied expression patterns (Figure 7). This means that some HECT genes, (for example *Bra005748*, orthologous to *UPL4*), may play important roles in plant adaption to adverse environmental stresses, an idea that was also supported by a previous study in apple [20]. These results are also consistent with our putative cis-regulatory element analysis (Figure 4), revealing the presence of important number of phytohormone-responsive or stress-related cis-regulatory elements in the promoter sequences of these HECT genes. Comparison of stress-responsive expression patterns between WGT-caused paralogous HECT members indicated that their functions may be conserved in some cases but diverged in others, a phenomenon that has been previously reported for RING finger protein genes [31]. Further studies should include more types of abiotic stress treatments and more *Brassica* species which would allow us to obtain a global view on the involvement of this gene family in stress tolerance of different *Brassica* species. The most prominent members, such as *Bra005748* or those highly responsive to stress treatments with high expression levels, can then constitute the gene candidates for further molecular mechanistic study as well as for use as targets in genetic engineering for improvement of stress tolerance in plants.

In conclusion, we conducted a comprehensive analysis of the HECT gene family in *B. rapa* and *B. oleracea*, including phylogenetic relationships, gene structures, motif composition within the HECT domain, additional protein domains, putative cis-regulatory elements, chromosomal location, synteny, Ka/Ks ratios, RNA-seq basesd gene expression, and qRT-PCR assays under salt and drought stress treatments. Ten and 13 HECT genes were respectively identified in *B. rapa* and *B. oleracea*. They were classified into seven groups along with the seven *Arabidopsis* HECT genes by phylogenetic analysis. RNA-Seq data analysis showed that the majority of them were constitutively expressed in all tested tissues. qRT-PCR based expression analysis showed that all 10 *B. rapa* HECT genes were responsive to salt and drought stresses. Thus, our study provides valuable information for further functional determination of each HECT gene across the *Brassica* species, and may help to select the appropriate gene targets for further genetic engineering and genetic improvement of *Brassica* crops.

## Figures and Tables

**Table 1:** Ka, Ks, Ka/Ks ratio and divergence time of the orthologous HECT gene pairs between *Arabidopsis* and *Brassica* species.

*A. thaliana* Genes	tPCK Chr	Block	*B. rapa* or *B. oleracea* Genes	Sub-Genome	Ka	Ks	Ka/Ks	Purifying Selection	Divergence Time (Mya)
*AT1G55860*	tPCK1	C	*Bra038022*	LF	0.0750	0.3214	0.2335	Yes	10.71~17.86
*AT3G17205*	tPCK2	F	*Bra022201*	LF	0.047	0.2672	0.1759	Yes	8.91~14.84
			*Bra021231*	MF1	0.0565	0.2557	0.2208	Yes	8.52~14.21
*AT3G53090*	tPCK6	N	*Bra040685*	MF1	0.0544	0.2827	0.1924	Yes	9.42~15.71
*AT4G38600*	tPCK4	U	*Bra010737*	MF2	0.0512	0.3403	0.1504	Yes	11.34~18.91
*AT4G12570*	tPCK5	P	*Bra029461*	LF	0.1828	0.7908	0.2311	Yes	26.36~43.93
			*Bra000779*	MF1	0.2205	0.7006	0.3147	Yes	23.35~38.92
*AT5G02880*	tPCK5	R	*Bra005748*	MF1	0.1163	0.3968	0.2932	Yes	13.23~22.04
			*Bra028860*	MF2	0.1078	0.3948	0.273	Yes	13.16~21.93
*AT3G17205*	tPCK2	F	*Bol036879*	LF	0.0473	0.2596	0.182	Yes	8.65~14.42
			*Bol009294*	MF1	0.0571	0.2513	0.227	Yes	8.38~14.06
*AT3G53090*	tPCK6	N	*Bol004417*	MF1	0.0506	0.2618	0.1932	Yes	8.73~14.54
*AT4G38600*	tPCK4	U	*Bol016067/8*	MF2	0.0552	0.3284	0.1584	Yes	10.95~18.24
*AT4G12570*	tPCK5	P	*Bol012097*	LF	0.1823	0.7928	0.2299	Yes	26.43~44.04
			*Bol030543*	MF1	0.2258	0.6826	0.3308	Yes	22.75~37.92
			*Bol003946*	MF2	0.2168	0.5982	0.3625	Yes	19.94~33.23
*AT5G02880*	tPCK5	R	*Bol015296*	MF1	0.0937	0.3733	0.251	Yes	12.44~20.74
			*Bol000770*	MF2	0.1066	0.3843	0.2773	Yes	12.81~21.35

## Supplementary Materials

The following are available online at https://www.mdpi.com/2073-4425/10/5/400/s1, Figure S1: Schematic diagram of HECT gene structures in the encoded genome of *Arabidopsis thaliana*, *Brassica rapa* and *Brassica oleracea*, Figure S2: Sequence logos for the 15 most conserved motifs identified among the HECT domains of *Arabidopsis thaliana*, *Brassica rapa* and *Brassica oleracea* HECT proteins shown in Figure 2, Figure S3: Ka/Ks annotated evolutionary tree of the HECT proteins genes from *Arabidopsis thaliana*, *Brassica rapa*, and *Brassica oleracea*, Figure S4: Expression values of *Arabidopsis thaliana* HECT genes in 47 developmental tissues, Figure S5: Expression patterns of *Arabidopsis thaliana* HECT genes in shoots (16 days after germination) under salt (A) and drought (B) stresses, Figure S6: Protein sequences of all HECT genes analyzed in this study, Table S1: List of identified *Brassica rapa* (*Bra*-) and *Brassica oleracea* (*Bol*-) HECT genes and their related Gene Ontology (GO) terms and chemical or physical information, Table S2: Primers used for quantitative real-time PCR (qRT-PCR).
