# Supplementary material for: Comprehensive Genomic Survey, Characterization and Expression Analysis of the HECT Gene Family in Brassica rapa L. and Brassica oleracea L
Source: Genes (Basel). 2019 May 27;10(5):400. doi: 10.3390/genes10050400 (PMC6562426; doi:10.3390/genes10050400)
Supplement: Supplementary file 1 [file genes-10-00400-s001.zip › Supplementary Figures S1-S6.pdf]

Supplementary Information

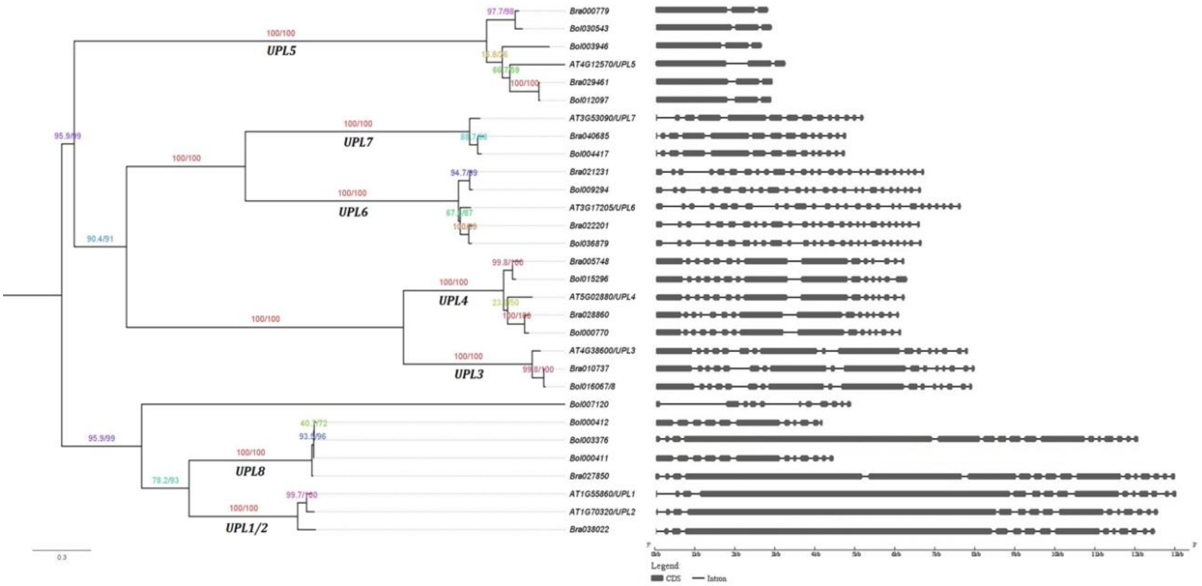

Figure S1. Schematic diagram of HECT protein gene structures in the encoded genome of *Arabidopsis thaliana*, *Brassica rapa* and *Brassica oleracea*. The maximum likelihood phylogenetic tree is provided on the left side of the figure, followed by the exons–introns on the right side, which are represented as grey boxes and black lines, respectively. The scale bar at the bottom (right side) indicates the lengths (Kb) of DNA sequences.

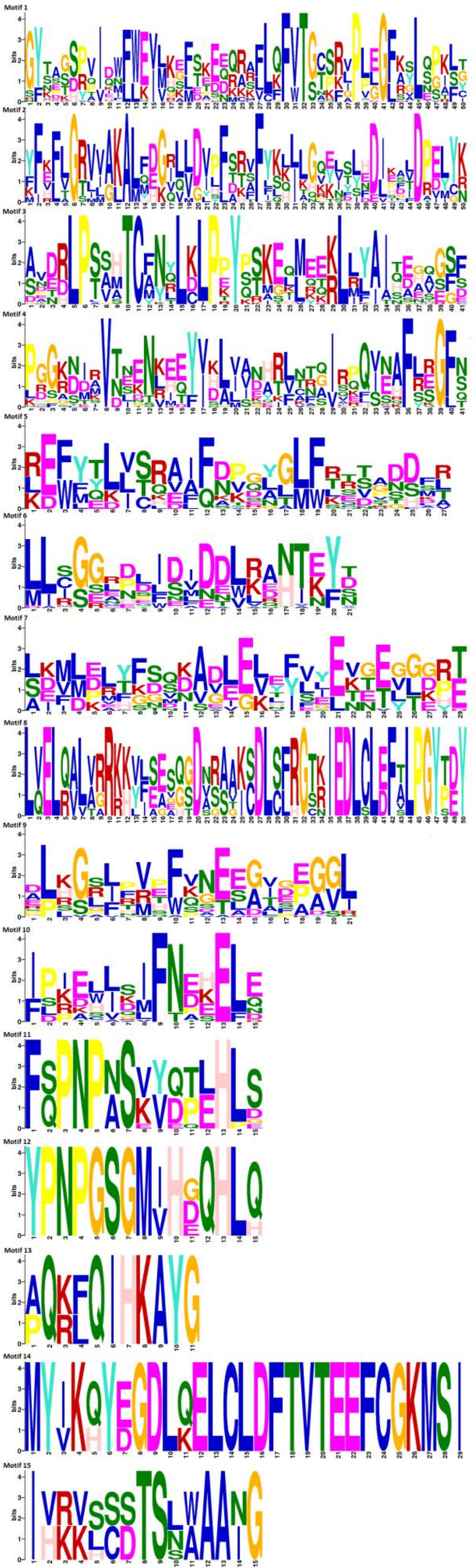

Figure S2. Sequence logos for the 15 most conserved motifs identified among the HECT domains of *Arabidopsis thaliana*, *Brassica rapa* and *Brassica oleracea* HECT proteins shown in Figure 2.

Ka/Ks annotated evolutionary tree

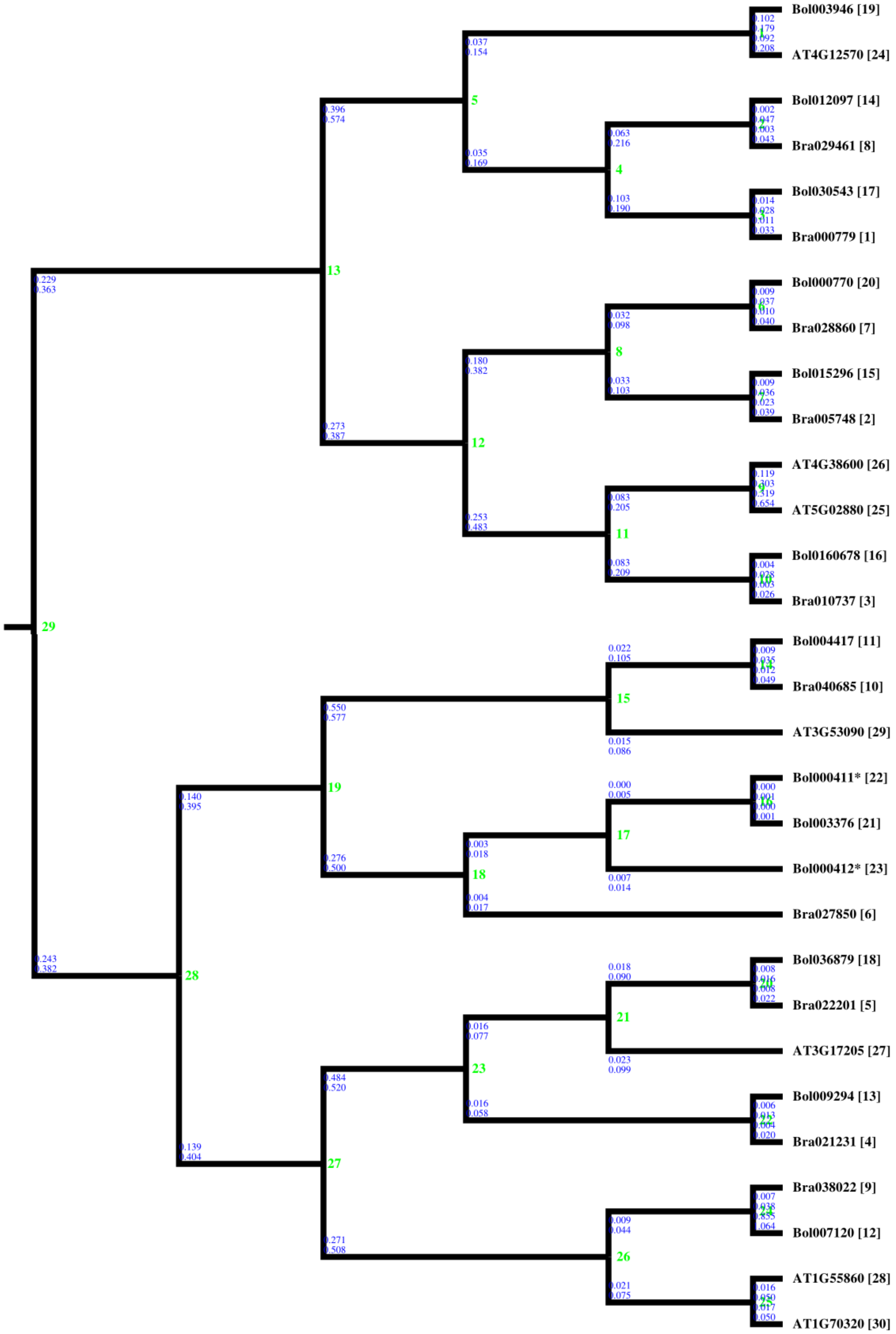

Figure S3. Ka/Ks annotated evolutionary tree of the HECT proteins genes from *Arabidopsis thaliana*, *Brassica rapa* and *Brassica oleracea*. The cDNA sequences and amino acid sequences of the HECT proteins from *A. thaliana*, *B. rapa* and *B. oleracea* were used to estimate the nonsynonymous (Ka) and synonymous (Ks) substitution rates and their ratio (Ka/Ks) for each node/branch using the online Ka/Ks Calculation tool at http://services.cbu.uib.no/tools/kaks. Twenty nine nodes are shown in green. The Ka and Ks values in each node and branch are marked in blue. All branches have Ka/Ks ratios less than 1.0, except *Bol000411* and *Bol000412* (marked in red) where both Ka and Ks equal zero.

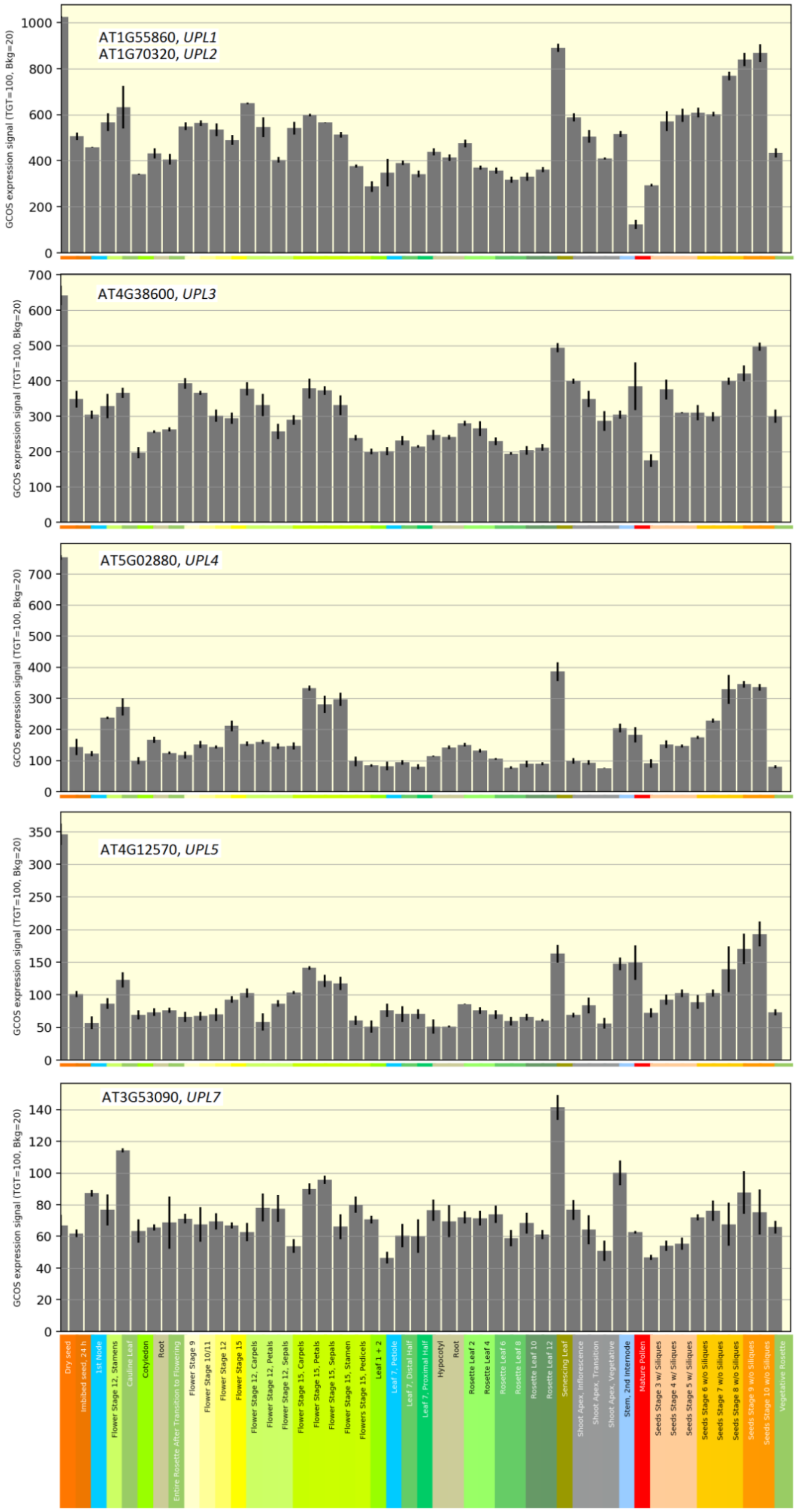

Figure S4. Expression values of *Arabidopsis thaliana* HECT genes in 47 developmental tissues. Charts of expression values were obtained from TAIR database (https://www.arabidopsis.org/) using the Arabidopsis eFP Browser. *UPL1* and *UPL2* share an identical chart, while *UPL6* is absent in the database. The 47 developmental tissues are indicated on the bottom with varied colors. GCOS, Affymetrix GeneChip Operating Software.

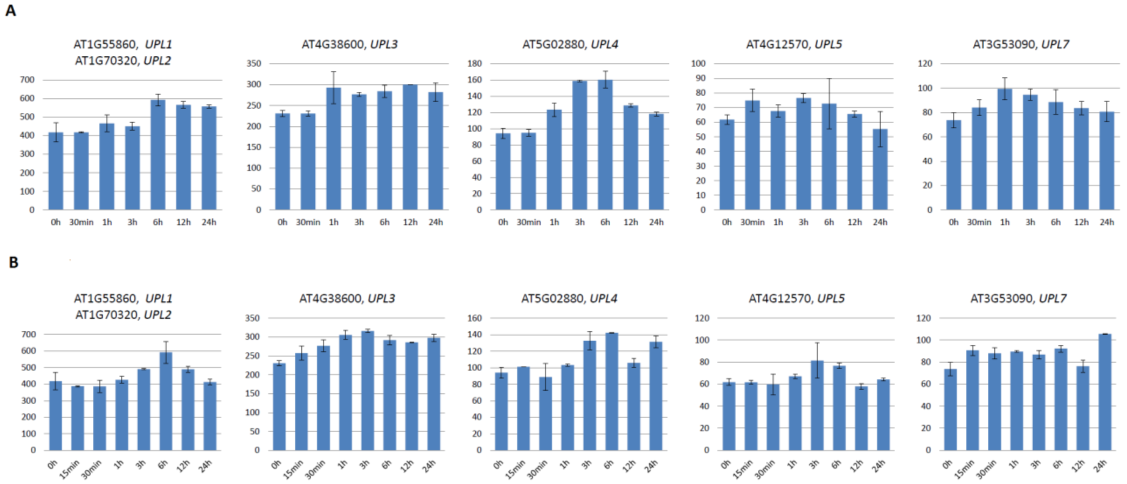

Figure S5. Expression patterns of *Arabidopsis* HECT genes in shoots (16 days after germination) under salt (A) and drought (B) stresses. Expression data were obtained from TAIR database (https://www.arabidopsis.org/) using the Arabidopsis eFP Browser. *UPL1* and *UPL2* share an identical set of data, while *UPL6* is absent in the database. The Y-axis values represent the expression levels generated by Affymetrix GeneChip Operating Software (GCOS). Salt stress was performed by transferring the plant in a media containing 150 mM NaCl. Drought stress was performed by exposing the plant to the air stream for 15 min with loss of approximately 10% of fresh weight.

Figure 6. Protein sequences of all HECT genes analyzed in this study. Green indicates the sequence has been revised by authors. Star (*) indicates the partial-length sequence.

>Bra000779
MTLSRYSDNPKRKLDDNGVDNNILHKMKRHEVDPDHTSPSQQPFNPWRSDENSPQNRYFS
DDCSSSSSSSTESSTRSESTRLQLFVRMVSGGKTIVIQADKSDTVENLHKRIEVKTKIPV
NEQRVIYKGKQLQDERSLSYYSIAQDSSLHLVGRMRSTDHPVACRVVDDIMYIVSLMHRG
GGGGGENNNLPRGMSINEKLSSFFSTIPTEAGGDDESSYIESTEKYLTIFTNSSVPAALV
MLYTSPLEANKSCGESSIKLFLNSCLALPEEQQGCCLPVVLEFCRLLRRVCPHNSLYGSC
RNTLGSLLETVQELVDKSGIEFTSLHYRLLTVGEEILPCLSELADLMEQQLYEDNLGPSL
YDVQKFSSFWRPLRYAIDFQLSSLIPIALPLRSTVLEAEVGRICQIFGRLLTTMHICMLR
IESSLGGRAVANTEAVALKWGQYLNILKIVNSMSELYQGGKEKVVRFINSRKVPFCALIL
KFVKRGDDHGWISEYREATTFECRRHLAMMLFPDGKEDYSEMHEMLIDRSQVLKESYVYI
SQASPAGLHGALFMEFKNEEATGPGVLREWFYLVCQEIFSPGGTLFLRSADDFRRFSPNP
ASKVDPLHLEYFKFAGRVIALALMHKVQVGVLFDRVFYLQLTNQTISLEDIKDTDRVIYN
SCKQILEMDPVFFDSNAGLGLNFVLETEELGKRETKELIKDGKSTAVDSKNREEYVKLLI
SERFVTSVSELVEKFSEGFSDILSVPIQSFFRHLDQEDFDGMLRGGENQISVDDWKAHTE
YNGFKETDRQIDWFWKILRKMTEEERRSVLFFWTSNKFIPLEGFRGLSSKLYIYRLHEAN
DRLPTSHTCFYRLCLPKYPTMGLMEQRLRFITQDHVSSSFGKW
>Bra005748
MENRGQKRMEDVEELPADKRACNSQDSRSSSSGGSSVQSQSQEAANGASSGHETTDADMD
TSSSDSPSSHSDGEPDKEEEEEEDYGSCDSDDDDEEGEDPRHKALQDVQWGRSSEDQQKL
SSLVTRLSEEVDPSLQLTGLTELCEVLSFCTEDSLSTVMADTLSRVLVKLANHESNADIM
LLAIRAVTYLSDVYPRSVAFLVKHETLPALCQRLQAIEYLDVAEQCLQALEKISKDEPVA
CLNAGAIKAVLSYIDFFSTSLQRVAVSTVVNICRKLSSESPSPFMDAVPILCNLLQYEDR
QLVENVAICLTKIADQVSESPEMLDQLSSHGLIHQSIHLLNLNGRTTLSQPVYNGVIGLL
RKLSSGSILAFRTLYELNIGYRLKEIISTYDISHSVSSTHPNNTCSNQVHEVLKLVIELL
PSSPVEDNQLALEKESFLVNQPDLLQQFGADILPVMIQVISDIQLYGAKIWSERICFLWL
PISNPQADLLDVLAGILSRKVHHVVVVALQIAEALLGKYSDDFLNSFIKEGVYFAIEALL
NSGQQNQGSADGSEEHVPKETVKCLCQSFERSSSSSSQTCKIEKDSVYILATRIKESFFG
PEVFDSQKGLTDVLQNLKHLSAALDDLMTEPIDAHALHDEKFFSVWSQIMERLNGRESVS
TFEFTESGVVKALTNYLSNRLHQRKFSKGDSECDSLPFVGNRFEVFTRSLWSDGEATSSV
LIKNLQNSLSSLENYPIVLSQFLKQRNSFATVPNGRSISYPILRVRFVKAEGETCLRDYS
QDLVTVDPLCFLDAVDQYMWPKVQLEPLYSVEEKDQAMECPSSQLESTSISCQGESSTHM
EIDSPNASQLQGSQEEDQEQLPDSGEDNTSSSEEEDALPEEDALPRLLFRLEGLELDRSL
TVYQAILLHKLKSGSETTNDSKLSGSHTITYERAPQLAESDENLFPLGFMDNDEYHPFLS
FLFAQRLDLRHKATNPPAYDMLFLLKSLEGMNRFLFHLICHERINAFGEGRLENLDDLRV
QLRPVPYAEFVSSKLTEKLEQQLRDSFAVSTCGLPPWFNDLMGSCPFLFSFEVKTKYFRL
AAFGSQKVHHHPQHLSSEGRPVTGSLPRKKFLACRETILESASKMMELHGNQKVVIEVEY
SEEVGTGLGPTLEFYTLVSRAFQNPDLGMWRCDRSSFAGKPKEDSGFLVAPSGLFPRPWS
DTSAAFPDVLQKFVLLGTVVAKALHDGRVLDIPFSKAFYKLIIGQELSSFDIHFIDPELC
KTLVELQALTRRKKVFSESQTDARAAKCDLSFRGTNIEDLCLEFVLPGYTDYVLALHSAN
DMVNLDNLEEYIKAIVNATICNGIQKQVEAFRSGFNKVFPIEHLGIFNEEELETLLCGER
DLFNLLEILHEFDKDQQRAFLQFVTGCPRLPPGGLASLNPKLTIVRKRGSDSSETDLPSV
MTCANYLKLPPYSSKEKMKEKLIYAITEGQGSFHLS

>Bra010737
METRSRKRAEATSTAPSSSSSSPPPPPSSGPTTRSKRARLSSPSSSSAAA
TAPSSSTRSRSSRSTTATVAVTPMDTSTESSGFHRGGGRGNRGNDNTNS
DKGKEKEHEVRIRDRERDRARQQLNMDAAAAAAAAADEDDDNDSEDGNG
GFMHPNMSSASSALQGLLRKLGAGLDDLLPSSGIGSGSSSHLNGRMKKV
LAGLRSEGEEGKQVEALTQLCEMLSIGTEDSLSTFSVDSFVPVLVGLLN
HESNPDIMLLAARALTHLCDVLPSSCAAVVHYGAVSCFVARLLTIEYMD
LAEQSLQALKKISQEHPTACLRAGALMAVLSYLDFFSTGVQRVAVSTAA
NMCKKLPSDASDYVMEAVPVLTNLLQYHDAKVLEYASICLTRIAEAFAS
SPDKLDELCNHGLVTQAATLISASNSGGGQASLGVSTYTGLIRLLSTCA
SGSPLGCRTLLLLGISSILKDILSGSGVSANASISPALSRPADQIFEIV
NLANELLPPLPEGSISLPTSANALVKGSGQKKSSPSTSGKQEDSPKVSP
REKLLSDQPELLQQFGLDLLPVLVQIYGSSVNGTIRHKCLSVIAKLMYF
STPEMIQSLIGDTNISSFLASVLAWKDPQVLVPALQVAEILMEKLPETF
SKVFVREGVVHAVDQLVLVGKPSSHASTDQENDCVPGSARSRRYRRRSS
NANSDGNQSEELKNSVSASIGANHNSMESPTASFMLRETVSSCAKAFKD
KHFPSDGGEFDVGVTDDLLHLKNLCTKLTAGTNDHKVKGKGKSKASGPC
LGDFSASKEEYLIGIISEILGELSKGDGVSTFEFIGSGVVAALLNYFSY
GYFSKEKISEVDLPKLRQDGLRRFTAFLEIALPSDGNEGKIPPMTVLIQ
KLQDALSSLERFPVVLSHPSKSLSGSARLSSGLSALAHPLKLRLCRAPG
EKALRDYSSNIVLIDPLASIAAVEEFLWPRVQRSESGVKPAAPVGNTEP
GTLPSGAGVSSPSSSTPASTTRHSSRSRSAIKIGDASKKEPVHEKGTSS
SKGKGVMKPAQPDKGPQTRSSAQRKAVLDKDTLMKPASGDSSSEDEEMD
ISPVDMDDALVIEEEDISDDDEDDDDEDVLDDNLPMCTPDKVHDVKLGD
AVDDEGAGLAPSGRQMNSALAGSSGTATARGSNSTDAGIGNLYGSRGAL
SFAAAAMAGLGAASGRGIRGSRDLHGRTLNRSSDESSKLMFTAGGKQLS
RHMTIYQAVQRQLMLDEDDDDRLGGSDFISSDGSRLNDIYTIMYQMPDS
QANRLSAGGASSTTPSKSTKSATTNASVEAQSYRASLLDSIVQGKLPCD
LEKSNSTYNVLALLRVLEGLNQLGPRLRAQTVSDRFAEGKITSLDDLNT
TAAKVSHEEFINSKLTPKLARQIQDALALCSGSLPSWCYQLTTACPFLF
PFQTRRQYFYSTAFGLSRALNRLQQQGADGSGSTNEREMRIGRLQRQK
VRVSRNRILDSAAKVMEMYSSQKAVLEVEYFGEVGTGLGPTLEFYTLLS
HDLQKVSLGMWRSNSGDKLSMQTDRDEIQDGKSAAARDRDIVQAPLGLF
PRPWPSTADVSEGSRFHKVVEYFRLLGRVMAKALQDGRLMDVPLSTAFY

KLILGQELDLHDVILFDAELGKTLQELRVLVGRKHYLEAGGGDNSSGIS
DLCLRGSRIEDLCLDFTLPGYPEYILRPGDDIVDINSLEDYISLVVDAT
VKRGVARQIEAFRSGFNQVFDIKSLQIFTPSELDYLLCGRRELWEAETL
VEHIKFDHGYTAKSPAIIFLLEIMGELTADQQRAFCQFVTGAPRLPPGG
LAVLNPRLTIVRKLSSTSNAAANGTGASETADDDLPSVMTCANYLKLPP
YSTKEIMYKKLLYAINEGQGSFDLS
>Bra021231
MFFSGDPSTRKRVDLGGRSTKEGDARKLLEQTKMERNRRLLQKKHNSAAL
KIQKVFRGRRSTAIVRSKVRRDFCETYGDNCQNVDRHCFEPASSFLPQF
LFFFKAQSSGDFVMLVETCRLLRKFAHSSGDILSLFSGLDYSSEHNMVD
FRVKKLAFTCIEAIHQNRDRLRDQLLVTPEEATISTAILMDAMSLLLDP
KLPWVCKIVSYLQKRNIFKLVREIVTTAKEGSRSPTMNGSILSLESVLF
LVVPHVGREPCCCPVVDPRWSFSSMILTIPFIWQLFPNLKVVFANPSLS
QHYVHQMALCIQKDTHVLPIDTNTEFPGNACLLGNTLEIANVVLSQPEC
SLDMAIDIASVATFLLETLPPIKSSERESRHSSSEEDDDMLIDDVQELV
LNRTLEQQITTNAIDSRFLLQLTNILFHQVSGGTQSYDEDKEALAIATG
SSFLYAAFNILPLERIMTVLAYRTELVAVLWNYMKRCHENQKWSSMPSV
LAYLPADSPGWLLPLVVFCPVYKHMLTIVDNEEFYERGKPLSLQDIRLL
IIILKQALWQLLWVNPLTQHNSGISVSNDLSKKNRVDLIQNRVGVVIAE
LLSQFQDWNNRQQFTSSTDFQADTVSEYFITQAIVEGTRASYILMQAPF
LIPFTSRVKIFTTQLATARQSHGSEEIFAGNRFRIRRDHILDDAYNQMS
ALSEDDLRGPIRVTFVNELGVEEAGIDGGGIFKDFMEKITLAAFDVQYG
LFKETVDHMLYPNPGSGMIHGQHLQFFHFLGSLLAKAMFEGILVDLPFA
TFFLSKLKQKYNYLNDLPSLDPELYRHLIFLKRYKGDISDLELYFVILN
NEYGERTEEELLPGGKDMRVTNKNVITFIHLVSNHRLNFQIRQQSSHFL
RGFQQLIPREWIDMFNEHELQVLISGSADSLDIDDLRENTNYSGGYNAG
HYVIDMFWEVMKSFSTENQKKFLKFVTGCSRGPLLGFKYLEPAFCIQRA
GGSVSNEAVDRLPTSATCMNLLKLPPYQSKEQLETKLMYAISAEAGFDL
S
>Bra022201
MFFSGDPSTRKRVDLGGRSTKERDAPKLLEQTRMERNRRLLQKQQNAAAL
KIQKFFRGRRSVAIERSKVRREFCETYGNNCQNVDRNCFEPGSSFLRQL
LFFFKAQNSGDFVILVETCRLLKIFTHSNGDILSLFSGLDYSAEHNLVD
FRVKKLAFTCIDAIHHNRDRLRDQLLVTTEEASTSTAILMEAMSLVLDP
KLPWVCKTVSYLQKRHIFQLVRDIVITAKESPRGQTMIGSILSLERVLI
LVVTHVGREPCCCPVVDPRWSFSSMILTIPLIWQLFPNLKVVFANPNLS
QHYIHQMAFCIQKDTHVLPTDTSAEFPGYACLLGNTLETANVVLSQPDC
SLDMAVDIASVATFLLERLPPVKSSERESKQSSSEEDDMLIDDIPEFVL
NKTLEQQITTNAIDSRFLLQLTNVLFHQVSLGTQPYDEEKEARAIGTAS
SFLYAAFNTLPLERIMTVLAYRTELVIVLWNYMKRCHENQKWSSMPNLL
AYLPGDAPGWLLPLVVFCPVYKHMLMIVDNEEFYEREKPLSLQDIRLLI
IILKQALWQLLWVNPLTQPSTGKSVSNNLSKKNPVGLIQNRAGVVVAEL
LSQLQDWNNRQQFTSSSDFQADTVNEYFISQAIAEGTRANYILMHAPFL
IPFTSRVKIFTTQLATARESHDHGSQAIFARNRFRIRRDHILEDAYNQM
SALSEDDLRGSIRVTFVNELGVEEAGIDGGGIFKDFMEKITRAAFDVQY
GLFKETVDHMLYPNPGSGMVHDQHLQFFHFLGTLLAKAMFEGILVDIPF
ATFFLSKLKHKYNYLNDLPSLDPELYKHLIFLKRYKGNIAELELYFVIL
NNEYGERTEEELLPGGKDMRVTNENVITFIHLVSNHRLNFQIRQQSSHF
LRGFQQLIPKECIDMFNEHELQVLISGSADSLDIDDLRQNTNYTGGYNA
GHYVIDMFWEVLKSFSTENQKKFLKFVTGCSRGPLLGFKYLEPAFCIQR
AAGSASNEAVDRLPTSATCMNLLKLPPYQSKEQLETKLMYAISAEAGFD
LS
>Bra027850
MATGRSASMPSRLRQLLSCEGSIGPSIRRDAEPPPEIKSFIEKVIQSPLS
DIAIPLSGFRWEYSKGNFHHWRPLFLHFDKYFKTFLSTRNDLLLSDHIL
EDEDPFPKYSLLQILRVMQIIILENCPNKSTFDGLEHFKLLLASTDPEVL
IAALETLSALVKISSSKLHRSGKLIGCGSVNSFLLSIAQGWGSKEEGLG
LYSCVVANERNQEEGLTIFPSDLDNNHKEADFRIGSTVYFELRGHSAQS
IHHGTSSASSSSSRVIQIPDLHLRKEDDLVLLKECIEHYNVPPDLRFSL
LTRVRYAHAFRSSRICRSYSRICLLAFIVLVQSSDAQEELASFFANEPE
YTNELIRIVRSEEPIPGTIRTLAMLALGAQLAVYSASHDRARILSRSSI
SFAVGNRMILLNVLQKAVLSLKISSDPSSIAFVEALLQFYLLHIVSSSS
SGSTIRGSGMVPTFLPLLEYTDPSHLHLVYLAVKALQKLMDYSSSAVPL
LRDLGGVELLSQRLELEVHHILKLTGENNSDMVVGESLDINGDQLLSRK
RLIKVLLKSLGSSTYTPGNANRSQSSQESTLPATLSLIYRNADKFGGDI
YYSAVTVMSDLIHKDPTSLTSLFEMGLPEAFLSSVVSGVLPSSKAIACI
PNGLGAISLNSKGLETVKETSALRFLVDVFTSKKYVLAMNEAIVPFTNA
VEELLRHVSSLRATGVEIIIEIVDRIACFGESGSSSSSVSTAMEMDSDV
KNINGVSHEQFVQLCIFHLIILLHRTMENAETSRLFVEKSGIEALLKLL
LRPSIAQSSEGVSIALHSTIVFKGFTQHHSASLARAFCSSLRDQVKKAL
GGLQDLSGTFLLDRKLSPSCGIFSSLFLVEFLLFLAASKDNRWMTALLS
EFANGSKDVLENTGLLHREILWHIAVYESGTLESPNTSPESEQTELSSN
GTEGQRFSSFRQFLDPLFQRRTSGWSAESQFFDLINLYRDLGRASTGFQ
PRPISDGPSSSSDASGNRELERDGSYYTSCCDMVKSLSFHITYIFQELG
KAMLLQSRRREDTVNVSPSSKLVASSFASISLDHLNFEGHEIAAEASRS
TKCLYLGKVVDFIDAVLLDRPDSCNPVLINCFYGRGVIQTVLTTFDATS
QLLFSINRSYSSPMETDDVKGKQDEKEDTDHAWIYGPLAGYGKLMDHLV
TSSFILSPSTKHLLVQPVENGNIPFPQDAETFVKILQSIVLKAVLPVWT
HPLFTECSYDFVTALISIIKHVYSGVELKSLAGSSSARVTGPPPSETTI
STIVEMGFSRSRAEEALRQVGSNSVELAMEWLFSHPEEIQEDDELARAL
AMSLGNSESDTKENVVDETREQIEAEIVSLPPVEELITTCTKLLQMKEP

LAFPVRDLLVLICSENNGEHRSGGICCLLSRIKDCCPVFDDTKNNLLSA
LLHVLALILHEDAGSREVALKAGTVRLVCDVLSKWDSGSIDKEKYNVPK
WVTTGFLAIDRLLQVDQKLNTELIEELQKGETSLAIDESKQDKLQSVFG
SPQLVDADDQKKLIEIACTCIRNQLPSETMHAVLQLCSTLTRKHSVAVC
FLDFGGVQGLLSLPSNSLFPGFDSVAASIIRHVLEDPQTLQQAARSICQ
VEMVGERPYIVLVKEKEKSKDKEKDKDKDRADKEKSQTSNEVATTTPPG
STKAKVYRKPPQSFIGVVELLLDSLCNFVPPPKDDMVEGDSTSADMDID
HASTKGKGKAVATTPAEKKAISQGMSASLAKIVFILKLLSEMLLMYSSS
IHIILRRDAEIKSLRGPQQKGGQVGGIFHHILRKFIPYSRVMKEKKSES
DWRQKLASRANQFLVGASVRSAEARKRIFSDISSIFNDFIGTSNGLRPP
VNEIHVLIVLLNDMLSARSPTGSHISSEASNTFVDVGLVKSLTRTLEVL
DMSASLAKIVFILKLLSEILFMYSSSIHIILRRDAEIKSLRGPQQKGGQ
VGGIFHHILRKFIPYSRVMKEKKSESDWRQKLASRANQFLVGASVRSAE
ARKRIFSDISSIFNDFIGTSNGLRPPVNEIHILIDLLSDMLSARSPTGS
HISSEASNTFVDVGLVKSLTRTLEVLDLDNVESPKTVTGIIKVLELVTK
EHAYSADSSSKNETANNSSDQIQSGRGDTTADASEAGEIMLRSNHDSMT
ADHAENFGGSHTFVGSEDVTDDMEHDQDLDEGFAAGVDDYMQEEPEDAR
GLENGIGSMGIEFEMHTHVPENLDEEEDEEMSGDEGDEIDEDENDNEEE
DEEDNDLEEEEEEDVHHLPHHDTDQDDHELEDDEFEEEILVEEDEEDED
DEDRVILRFEDGINGLNVLDHLEVLRDHRFSDETLHVMPVEVFGSRRQG
RTTSIYSLLGRTGDGATPSQHPLLSGSPSLQGSQSQTESTHDHTVGGRD
SNGSSSSRLDAIFRSLRNGRQGHRLNLWADDSQQIVGSGASTVPQGLED
LLVSQLRRPGSDNPSDQNPSPLEPQSQAESGQPQEATVRPEIPDENATG
NGGANVSAPSIVSPVASAPPDTRATATDSVSSSRSQSVEMQYDLIPDENA
TGNGGANVSAPSIVSPVASAPPDTRATATDSVSSSRSQSVEMQYDPNDS
TVRDVEAVSQESGGSGATLGESLRSLDVEIGSADGHDDGAERHDVQPAM
RSRRANLSVVPSSTGREASLYSVTEVPENSGHEAEQDNPPEEQPVNRDV
ASSSIDPAFLDALPEGLRAEVLSAQQGQAPEPSSNEQQNSGDIDPEFLA
ALPADIRAEVLAQQQAQRVHQSHELEGQPVEMDTVSIIATFPSELREEV
LLTSDDAVLANLTPALVAEANMLRERFAHRYHNRALFGMHPRHRRGEAS
RRGEGVISGNEGIASRRSAAKVIEAAGAPLVNTEALQAMIRVLRIVQPL
YKGPLQRLLLNLCSHGETRFSLVNTFMDMLMLDTRKPVNYSSVSEPPYR
LYACQSNVTYSRPQHFDGVPPLVSRRVLETLTYLARNHIYVAKILLQSR
LSLPSLQGSVPSDKARGKAVVVSDDHMSRTQQEPESVAFALLLSLLNQP
LYLRSVAHLEQLLNLLEVIIDNAERKSESADGSDGSASEQQSTHQALEV
ENNAENHDMVSGTAGTVTKPIVSSGSSSYRAESECDVHTVLLNLPQSEL
CLLCSLLAREGLSDNAYTLVAEVLKKLVAIAPSHCHLFITELANAIQNL
TRSAISELHMFGEAVKTLLSTTSSDGSGVLRVLQALSSLVDSLLITKEK
NSEEHVAVFCQLSNINLALEPLWLELSNCICKIEGHSDSASASTTSPTT
STSSATTRGAGVSQSLPAGAQNMLPYVESFFVTCEKLHPSSQSCDISVP
MAASDVEEQPKGPGPSSSSKVDEKYGSFIKFSERHRKLLNAFIRQNPAL
LEKSFSLMLKVPRFIEFDNKRAYFRSKIKHQHDHHHSPLRISVRRAYIL
EDSYNQLRMRSTQELKGRLTVHFQGEEGIDAGGLTREWYQLLSRVIFDK
GALLFTTVGNDSTFQPNPNSVYQTEHLSYFKFVGRVVGKALFDGQLLDV
HFTRSFYKHILGVKVTYHDIEAIDPDYYKALKWMLENDISDVLDLTFSV
DADEEKLILYEKTEVTDHELIPGGRNIKVTEENKHEYVDLIAEHRLTTA
IRPQINAFLEGFSELILKDLISIFNDKELELLISGLPDIDLDNLRANTE
YSGYSPGSPVIQWFWEVVQGLSKEDKARLLQFVTGTSKVPLEGFSSLQG
ISGAQKFQIHKAYGSANHLPSAHTCFNQLDLPEYPSKEHLQERLLLAIH
EASEGFGFG
>Bra028860
MENRGQKRMEVVGEPPADKRACNSQDFTSGASSAQAQQANGNTDADMDTS
SSASPSSRSDGEQDREEEEEEESDYGSCDSDDEDPRRRVLQRYQRGRST
GDQLKLKSLASRLSEENDPSLQLTGLTELCEVLSFCTEDSLSIVMADLL
SRVLVKLAKHESNADIMLLAIRAVTYLSDVYPRSVAFLVKHETIPALCQ
RLLTIEYLDVAEQCLQALEKISRDQPVACLNAGAIMAVLSYIDFFSTSI
QRVAVSTVVNICRKLPPEPPSPVMDAVPVLCNLLQYEDRQLVESVAICL
TKIADQGVIGLLRKLSSGSTLAFRTLYELNIGYRLKEIISTYDISHSVS
STQPIHPCSNQVHEVLKLVIELLPASPVGDNQLALEKESFLVDQPNLLQ
QFGADMLPVMTQVLKSGASVYVSYGCLSAIHKLTCLSKSDDLVELLNNA
NISSVLAGIFSRKDHHVVVVALQIAEVLLEKYRDAFLNSFIKEGVFFAI
AALLTSDRGQQINPVSGFIQGSVPKEIVKCLCQSFEGSVSSSSQTCKVG
NDSVYILATRIKESFFGPEVFDSQKGLTDVLQNLKNLSAELNDLVTVPV
DAHVLHGERFFSIWNQIMARLKERESVSTFEFTESGVVKALANYLSNGL
YERKLNKGDPECDSLPLVGNRFEVFTRLLWSDGEATPSALIQKLQNSLS
SLENYPIVLSQFLKQRNCFAAIPNGRCISYPVLRVRFAKAEGETCLRDY
SPNFVTVDPLCYLDAVSQCLWPQVNLEPLNSVEAKDQAIECQSSQLQST
SISCQGESSSHMEIDCPNASQLQGSQEEEDQYHLIDSGEENSSSSKEED
VRPRLLFRLEGLELDPSLTVYQAILSHKLKSENETTNDSKLSGHHTITY
ERAPQLAVSHENLFPLRSMDNDEHHPFLSYLFAHRLGLRHKGTSPPEYA
ILFLLKSLEGMNRFLFQLICHERINAFGEGRLESLDDLTVQVRPVPYAE
FVSSKLTEKLEQQLRDSFAVSPCGLPPWFNDLMASCPFLFSFEVKSKYF
RLAAFGPQQVHNQPQHLGSSNVHGSLPRKKFLACREKILESAAKMMELH
GTQKVAVEVAYSEEVGTGLGPTLEFYTLVSRAFQNPDLGMWRSDPSSLA
GKPMVPPSGLFPRPWSATSAAFPGVLQKFVLLGTVVAKALQDGRVLDIP
FSKTFYKLILGQELSSFDIHFVDPELCKTLVELQALARRRKVISESQSD
VRAAKCDLSFRGTKIEDLCLDFSLPGYTDYVLSPRFANDMVNLGNLEEY
VKAIVNATVCNGIKKQVEAFRSGFNKVFPIEHLKIFNEEELETLLCGER
DLFNMNEVLDHIKFDHGYTSSSPPVQNLLEILHEFHKEQQRAFLQFVTG
CPRLPPGGFASLSPKLTIVRKHGSDSSETDLPSVMTCANYLKLPPYSSK
EKMKEKLIYAITEGQGSFHLS

>Bra029461
MTLRRSTAADSPISHRSPLAVAGTDNHKRKLDDYAADYGLDLLQKMKRHE
VDADRISPASQQKPLTSGENYRSLYTPGECSSSSSPSSSSSLPESWTRS
ESTRLQLFVRMMSGGKTIVIHADRNDTVEHLHHRIQLKTQIPVTEQRVI
YKGKQLQFEHTLNHYSIEQDSSLHLVGRMQSTEYPVACQTVDEIMSTIS
RMHRGENLYGGRTNINDKLVKFFARIPAESNRSSAKYLKIFSNSSVPAA
LVMLFVSPLEINKACGKSSIKLFLNSCVALPVHQQNSCLPVVLEFCRLL
RGACPDNKLYASCRNTLGSMLDLVCKSDEFQFRLFTIGEEIYPCVTELA
DIIVRELVENTGPGLSEVQKFSSFWQPLKRAITAQLPCLFPVAMPLRNT
VMEAEIGKLFQIFRRLMTVMDICMTRIESSLGNRGVANTEAMSATWSQY
LAVLKIVDSMCELYQGAKEQLASLLNARKVSFSALVLKFAKRGDDDHQW
IFDYKEATTFESRRHLAMLLFPDVKEDYDEMHEMLIDRSNLFAESFEYI
SGATPGSLHSGLFMEFKNEEATGPGVLREWFYLVCQEIFNPRNALFLRS
ADDFRRFSPNPASKVDPLHLSYFEFTGRVIALALMHKVQVGVLFDRVFF
SQLAHPEISLEDIKDTDRVMYNSCKQILEMDPAFFDSNAGLGLTFELET
EELGKRETVELLPDGRSIAVNSENREQYVKLLIKQRFAASISQHVERFS
KGFSDILSDSVPAFFKRIYLEDFDGMLRGGENPISIDDWKAHTEYNGFK
ETDRQIDWFWKIMKKMTEEERRSVLFFWTSTKFIPVEGFRGLSSKLYIY
RLHEANDRLPTSHTCFYRLCLPKYPTMGLMEQRLRFIAQDHVSSSFGKW
>Bra038022
MPSQVQPPKIKSFINSVTAVPLDQIQEPLSCFHWDFDDKGDFHHWVDLFN
HFDTYFEKHIKARKDLHVEQQDSEDESTPPLPKDALLQILRVIRVVLDN
CTNIHFFTSYEQHLSLLLASTDTDVVEACLQTLASFFKRQNDIYFIRDA
SLNSKLFSLAQGWGGKEEGLGLTSCATTENTCDLVSHLLGSTLHFEFYA
SGESSTELPGGLQVIHLPDVSLRAESDLELLNKLVTDHNVPPSLRFVLL
TRLRFARAFSSLSTRLQYTRIRLYAFILLVQASGDTQKVVSFFNGEPEF
VNELVTLLSYETTVPEKIRLLCLLSLVALSQDRTRQTTVLTAVTSRGLL
SGLMQKAIDSVLCNTSKSSLAFAEALLSLVTVLVSSSSGCSAMQEAGLI
PILVPLIKDTDPQHLHLVSTAVHILEAFMDYSNPAAALFRDLGGLDDTI
FRLKLEVSRTEDNVNEKVCGSDSNGRASHVLGDSLNRPDTEQLPYSEAL
ISYYRRLLLKALLSAISLGTYSPGNTNLYGSEESLLPECLCIIFRRAKY
FGGGVFSLATTVMSDLIHKDPTCFNTLDSSGVTSAFLDAISDEVICSAQ
AITCIPQSLDALCLNNSGLQAVKDRNALRCFVNIFTSSSYLRALTGDTP
SALSSGLDELLRHQSSLRTYGVDMFIEILNSMLIIGSGMEASTSVSADV
PTDAATAPMEIDADEKSLAISDEAEPSSAASPANTELFLPDCVCNVARL
FEIVLQNAEVCSLFVEKKGIDVVLQLLSLPVMPLSTSFGQNFSVAFKNF
SPQHSASLSRTVCSYLRERLKGTNELLGAIKGTQLLKLESAVQMTILRS
LFCLEGMLSLSNFLLKGTSSVIAELSAADADVLKELGLTYKQIIWQMAL
SSETKEDEKKSVDGGPDNSILASSSTVERESEEDSRNASAVRYTNHVSI
RRSTSQSIWRGGRDLSVMRSIESMHGRTRQAISRTRGGRTRRHLEAFNF
DSEIPPDLPGTSSSHELKKKSTEVLTVEILDKLNCTLRLFFTALVKGGF
TSANRRRIDGAPLSSASKKTLGNAIAKVFLEALNFDGNGVTAEHDIFLS
VKCRYLGKVVDDMASLTFDTRRRVCFTAMINSFYVHGTFKQLLTTFEAT
SQLLWTVPFSVTASDTENEKPGERNIWSRKTWLVDTLQIYCRALDYFVN
STFLLSPASTSQTQLLVQQEQASIGLSIELHPVPREPETFVRNLQSQVL
DVILPIWNHPMFPDCNPNFVASVTSLVTHIYSGVVDATQNQARGTNQRA
LPLQPDETIVGMIVEMGFSRSRAEYALRRVGTNSVEIAIEWLFANPEHT
VQEDDELAQALALSLGNASKTPKPVDVPLEEADPKEPSVDEVITASVKL
FESDDSMAFPLMDLFVTLCSRNKGEDRPKIVSFLIQQLKLVQVDFSKDT
GALTMLPHILALVLSEDDNTREIAAQDGIVTVAIDILTNFKLKSESESQ
ILAPKCISALLLILSMMLQARTRISSEFLEGNHGGSLEPSDYPQDSAAA
LKKVLSSDVAKEESKPDLESVFGKSTGYLTMEEGQKALLIACGLVKQCV
PEMIMQAVLQLCARLTKTHALAIQFLENGALSSLFNLPKKCFFPGYDTV
ASVIVRHLVEDPQTLQIAMESEIRQTLSGKRHVGRVLPQTFLTTMAPVI
SRDPVVFMKAVASTCQLESSGGRDFVIPSKEKEKPKVSSSEQGLPLNEP
LRISENKLHDGSGKCSKSHRRVPANFIQVIDQLIDIVLSFPRVKRQEDD
ETNLIAMEVDVPATKVKGKSKVGDPEEAEFGSEELARVTFILKLLSDIV
IMYLHGTSVILRRDTEISQLRGSNLPDNSPGNGGLIYHIIHRLLPISLK
NSVGSEVWKEKLSEKASWFLVVFCSRSSEGRRRIISELSSVLSVLASLG
KSSSSKSVLLPDKRVLAFAGLVYSILTKNSSSSNLPGCGCSPDVAKSMI
DGGIIKCLTSILHVIDLDHPDAPKLVTLILKSLETLTSAANTAEQLKSA
GSNETKGTDSNERHDSRGTSTEAEVDESNRNNSSLQQVTDAAENGQEHP
QISSQSEGGRGSSQTQAMPQEMRIEGEETILPEPIQMDFMGEEDDQIEM
NFHVENRAGDDGDDAMGDEEDDDEEGFDDIGPELEDDEDADLVADGARS
VMSLSGTDAEDPEDTGLGDEYNDDMIDEDEDDIHENRVIEVQWREALDG
LDHFQILGRSGGGNEFIDDFEGMNMGDLVTLQRPGFDRRRQADINSFHR
SGSQVHGFQHPLFSRPLRTGNTASVSASAGRNDISQFYMFDMPVIPFDQ
VPSNPFSDRLGGSGAPPPLTDYSVVDMDSSRRGVGNSRWTDIGHPQPSS
QSASIAQLIEEHFISNLRASALADSVVERETNSTEVQEQQHPSVGSESV
LGDGNDGGQQSEAHEMLNNNDNVDNPPDVTAGIFSQARANLASPVLLQP
LPMNSTPNEIDRMEVGEGDGVPIEQADVVAVDLVSTAQGQPDTSSSQNV
SGMGTPIPVDDPISNCQPSGDVHMSSDGAEGNQSVEPSLLSRDNNELSSR
EATQDASNDEQLAEGSLELDGRAPEANSIDPTFLEALPEELRAEVLASQ
QAQSVQPPTYEPPSVEDIDPEFLAALPPDIQTEVLAQQRVQRMAHQSQG
QPTDMDNASIIATLPADLREEVLLTSSEAVLAALPSPLLAEAQMLRDRA
MSHYQARSHSNRRNGLGYNRLTGMNRNVGVTIGQRDVSSFADGLKVKEM
EGDRLVDVEALKSLIRLLRLAQPLGKGLLHRLLFKLCAHRGTRANLVQL
LLDLIRPEMETSPSELAISNQQRLYGCQSNVIYGRSQLLNGLPPLVFRR
VLEVLTYLATNHSAVADMLFYFDSSLVSQLSKPKPSVCEGKGKETVTHV
TDSRNLEIPLVVFLKLLNRPQLLQSTSHLALVIGLLQEVVYTAASRIEG
WSPLSSLSEKSEEKPVGEEASSETRKDAKSEQVDEADKQSVARVKNCAD

IYNIFLQLPQSDLCNLCLLLGYEGLSDKIYLLAGKVIKKLAAVDVAHRR
FFAKELSQLASGLSASTVRELATLSNTEKMSHSTGSMAGASLLRVLQVL
SSLTSTIDDGNPGTEKETEQEEQNIMERLNMALEPLWQELSQCISMTEV
QLDHTSATTTVSSVNPGDHALGVTAPSPISPGTQRFLPLIEAFFVLCEK
IQTPSILHQDQANVTAGEVKESALSLSSKTSVDSQKKIDGSLTFAKFAE
KHKRLLNSFVRKNPSLLEKSLSMMLKAPRLIDFDNKKAYFRSRIKHQHD
QHISGPLRISVRRAYMLEDSYNQLRMRSLQDLRGRLNVQFQGEEGVDAG
GLTREWYQLVSRVIFDKGALLFTTVGNDATFQPNPNSVYQNEHLSYFKF
VGRMVAKALFDGQLLDVYFTRSFYKHILGVKVTYHDIEAVDPDYYKNLK
WLLENDVSDILDLTFSMDADEEKHILYEKTEVTDYELKPRGRNIRVTEE
TKHEYVDLVAGHILTNAIRPQINAFLEGFNELIPRELVSIFNDKELELL
ISGLPEIDFDDLKANTEYTSYTAGSPVIHWFWEVVKAFSKEDMARFLQF
VTGTSKVPLEGFKALQGISGPQRLQIHKAYGGPERLPSAHTCFNQLDLP
EYPSKEQLEERLLLAIHEASEGFGFA
>Bra040685
MDLNRKHKVSLRGASSEEMSRDALLAKVSQARELRSYARRANAAAVFIQR
VWRSYIVRKKAAFVIQDEWESSLSCHSDTLTKSWVSSRVLRPFLFFIRS
LCVQHQKIEPRDVQCMQTCFKILLESINSNDQGYNFCSLAVGTFEESRT
WLCQTRRLVSLCSFLLTECSYSQERIKDVLGVGALLLRILIVLTDPKSW
KIVSKQNFEDAESAGKMIVPFIGNSKSGYYTAVRRYIKSLTKNSDERLL
ITTSAVTLALRPFHARQPDSDGDNQPDTNLAVEEYVSLILTIPRLVCCL
PSVLIRALKHKSILMPSFHTILILRDKLLTRISEIEYSERQSCTTEIPS
VGWAMGNIVSLATVSENDFMDTQESNLEMFYVLYVHVIVTLTENLLSQV
EKVGTQDIHLDFEATPDDTEKGKSSVKISFVELLRPVCQQWHLAKLLAA
SGKETRIIADKDASTNSKKGSETLELFDVARFYSCMLRIFCVLNPVVGP
LPVLNMLSFSPGYIVSLWSSLENVLLSEDGCIPDESSHKPAKASWNTRS
PSEKKLKHVKNDGVNKWVNVLNKFSGKSPGPREHVESTSDQPGSSLIND
STDDGWDVQTLRGGPVGISKDVSCLLHLFCATYAHLLVVLDDIQFYEKQ
VPFMLEKQRRIASVLNTLVYNGLLRGTGPENRQLMDSAIRCLHLLYERD
CRHPFCPSTLWLSPGRTSRPPIAFAARTHEVLPTSDVFTSPSMGSVITI
TPHVFPFEERVHVFREFISMDKASRKMAGEVDAPGARSIEIVVRRGHVV
EDGFRQLNSIGSRLKSSIHVSFVNESGLPEAGLDYGGLSKEFLTDITKA
AFASEYGLFSQTTTSDRLLVPSPSARYLENGIQMIEFLGRIVGKALYEG
ILLDYSFSHVFIQKLLGRYSFIDELSGLDPELYRNLMYIKQYEGDLQEL
CLDFTVTEEFCGKMSIIELKPGGKDISVTNENKMQYIHAMADYKLNRQI
VPFSNAFYRGLTDLISPAWLKLFNAHEFNQLLSGGNHDIDVDDLRRNTK
YTGGYSDSSRTIKIFWEVMKGFEPSERCMLLKFVTSCSRAPLLGFKYLQ
PTFIIHKVSCDTSLWAAIGGQDVERLPSASTCYNTLKLPTYKRASTMRE
KLLYAITSNTGFELS
>Bol004417
MDLNRKHKVSLRGASSEEMSRDALLAKVSQARELRSYARRANAAAIFIQR
VWRSYIVRKKAAFVIQEEWESSLSCNSDTLTKSWVSSRVLRPFLFFIRSL
CVQHQKIEPRDVQCMHTCFKILLESINSNDQGYHFCSLAVGTFEESKTWA
CQTRRLVSLCSFLLTECSYSQERIKDVLGVSALLLRILIVLTDPKSWKII
SKQNFEDAETAGKMIVQFLGSSKSGYYRAVRKYIKSLTKNSDERLLITTS
AVTLALRPFHARQPTFVDDNQPDTNLAVEGYVSLILTVPRLVCCLPSALI
RALKHKSILMPSFHTILILRDKLLTRISEIEHSEKQSCTTEIPSVGWAMG
NIISLATVSENDFMDTQESNPEMFYVLYVHVIVTLAENLLSQVEKVGTRD
IHLDFEATPDDTEKGKSSVKISFVELLRPVCQQWHLAKLLAASGKETRVT
ADKDASTRSKKGSETLELFDIARFYSCMLRIFCVLNPVVGPLPVLNMLSF
CPGYVVSLWSSLESVLLPEDGCTADESSHKPAKASWNTRSASEKKLKHLK
NDGVNKWVNVLNKFSGKSPGPREHVESTVDQPGSSQANESTDDGWDVQTL
RSGPVGISKDVSCLLHLFCASYAHLLVVLDDIQFYEKQIPFTLEKQRRIA
SVLNTLVYNGLLRGTGPENRQLMDSAIRCLHLLYERDCRHPFCPSALWLS
PGRTSRPPIAFAARTHEVLPTNDVFTSPSMGSVITITPHVFPFEERVHVF
REFISMDKASRKMAGEVDAPGARSIEIVVRRGHVVEDGFRQLNSIGSRLK
SSIHVSFVNESGLPEAGLDYGGLSKEFLTDITKAAFATEYGLFSQTTTSD
RLLVPSPSARYLENGIQMIEFLGRIVGKALYEGILLDYSFSHVFIQKLLG
RYSFIDELSGLDPELYRNLMYIKQYEGDLQELCLDFTVTEEFCGKMSIIE
LKPGGKDISVTIENKMQYIHAMADYKLNRQIVPFSNAFYRGLTDLISPAW
LKLFNTHEFNQLLSGGNHDIDVDDLRRNTKYTGGYSDSSRTIKIFWEVMK
GFEPSERCMLLKFVTSCSRAPLLGFKYLQPTFIIHKVSCDTSLWAAIGGQ
DVERLPSASTCYNTLKLPTYKRASTMREKLLYAITSNAGFELS
>Bol007120
MNCRGRSPMNYDGQYTPVVTNMDKPRTLPPFLKNSRRPLVKSLIEMVKLV
DPKDSEAFLFSEHVLRSLNLISKVCRESGDHTVSPSQLILCDVDSIKFED
GNRENLFTFRQTGPSKWKPTIDRVHLNTHSKLLETRLANAKEQQLVKTQI
LLSDLMEQNDEHFARILLDVLHAETKTSTSDQMVISWVLILLRRLIISSN
PILEVFLVRDLALLLELLNLPSKELMMLVMHLLVSVLSHAKVEDESSKYS
LDELKPSDLQNLCIFFRKDSQTFNLVSTLRTTHSSACVVAFALSSNNIMD
VTFTPSFYKHMIGDDITYHDMKTMDHELFNEWEKLLQTMVTKESVQKYIE
EEAEYFMTGAIDDKLLAFLKGFHELIPPKYAAIFNAEELRDAMSGMPTIH
VEALKQTAIYVNYVGDDVVIQWFWEVLESFEHCDKAKFLGFVTGTQRVPS
DGLELTIVRSGATNRLPAGHTCAKQLDLPRYSTKEQLRDKLLKAIQGCDR
FCFG
>Bol009294
MFFSGDPSTRKRVDLGGRSTKEGDARRLLEQTRMERNRRLQQKQHNSAAL
KIQKFFRGRRSMAIERFKVRRDFCETYGDNCQNVDRHCFEPASSFLPQFL
FFFKAQSSGDFVMLVETCRLLRQFAHSCGDILSFFSGLDYSTEHNTVDFR
VKKLAFTCIEAIHQNRERLRDQLLVTPEEATISTAILMDAMSLLLDPKLP
WVCKIVSYLQKRNIFKLVREIVTTAKEGSRSPTMNGSILSLESVLFLVVP

HVGREPCCCPVVDPRWSFSSMILTIPFIWQLFPNLKVVFANPSLSQHYVH
QMALCIQKDTHVLPIDTNTEFPGYACLLGNTLEIANVVLSQPECSLDMAI
DIASVATFLLETLPPIKSSERESRHSSSEEDDDMLIDDVQELVLNRTLEQ
QITTNAIDSRFLLQLTNILFHQVSGVTQSYDEDKEALAIGTGSSFLYAAF
NILPLERIMTVMAYRTELVAVLWNYMKRCHENQKWSSMPSVLAYLPADSP
GWLLPLVVFCPVYKHMLTIVDNEEFYERGKPLSLQDIRLLIIILKQALWQ
LLWVNPLTQHNSGKSVSNDLSKKNRVDLIQNRVGVVVAELLSQFQDWNNR
QQFTSSTDFQADTVREYFITQAIVEGTRASYILMQAPFLIPFTSRVKIFT
TQLATARQSHGSEEMFAGNRFRIRRDHILDDAYNQMSALSEDDLRGPIRV
TFVNELGVEEAGIDGGGIFKDFMEKITLAAFDVQYGLFKETADHMLYPNP
GSGMIHGQHLHFFHFLGSLLAKAMFEGILVDIPFATFFVSKLKQKYNYLN
DLPSLDPELYRHLIFLKRYKGDISDLELYFVILNNEYGERTEEELLPGGK
DMRVTNKNVITFIHLVSNHRLNFQIRQQSSHFLRGFQQLIPREWIDMFNE
HELQVLISGSADSLDIDDLRENTNYSGGYNAGHYVIDMFWEVMKSFSTEN
QKKFLKFVTGCSRGPLLGFKYLEPAFCIQRAGGSVSNEAVDRLPTSATCM
NLLKLPPYQSKEQLETKLMYAISAEAGFDLS

>Bol012097

MTLRRSTAADSPISHRSPLAVAGTDNHKRKLDDYAADYGLDLLQKMKRHEVDADRISPAS

QQKPLTSGENYRSLYTPGECSSSSSSSSSLPSESTRLQLFVRMMSGGKTIVIHADRNDTV

EHLHHRIQLKTQIPVTEQRVIYKGKQLQFEHTLNHYSIEQDSSLHLVGRMQSTEYPVACQ

TVDEIMSTISRMHRGENLYGGRTNINDKLVKFFARIPAESNRSAAKYLKIFSNSSVPAAL

VMLFVSPLEINKACGKSSIKLFLNSCVALPVHQQNSCLPVVLEFCRLLRGACPDNKLYAS

CRNTLGSMLDLVCKSDEFQFRLFTIGEEIYPCVTELADIIVRELVENTGPSLSEVQKFSS

FWQPLKRAVTAQLPCLFPVAMPLRNTVMEAEIGKLYQIFRRLMTVMDICMTRIESSLGNR

GVANTEAMSATWSQYLAVLKIVDSMCELYQGAKEQLASLLNARKVSFSALVLKFAKRGDD

DHQWIFDYKEATTFESRRHLAMLLFPDVKEDYDEMHEMLIDRSNLFAESFEYISGATPGS

LHSGLFMEFKNEEATGPGVLREWFYLVCQEIFNPRNALFLRSADDFRRFSPNPASKVDPL

HLSYFEFTGRVIALALMHKVQVGVLFDRVFFSQLAHPEISLEDIKDTDRVMYNSCKQILE

MDPAFFDSNAGLGLTFELETEELGKRETVELLPEGKSIAVNSENREQYVKLLIKQRFAAS

ISQHVERFSKGFSDILSDSVPAFFKRIYLEDFDGMLRGGENPISIDDWKAHTEYNGFRET

DRQIDWFWKIMKKMTEEERRSVLFFWTSTKFIPVEGFRGLSSKLYIYRLHEANDRLPTSH

TCFYRLCLPKYPTMGLMEQRLRFIAQDHVSSSFGKW

>Bol015296
MENRGQKRMEDVEELPADKRACNSQDSRSSSSGGSSVQSQSQEAANGASS
GHETTDADMDTSSSDSPSSHSDGEPEKEEEEEEDTDYGSCDSDDEEGENP
RHKALQDVQWGRSSEDQQKLSSLVTRLSEEVDPSLQLTGLTELCEVLSFC
TEDSLSTVMADTLARVLVKLANHESNADIMLLAIRAITYLSDVYPRSVAF
LVKHETLPALCQRLQAIEYLDVAEQCLQALEKISKDEPVACLNAGAIKAV
LSYIDFFSTSLQRVAVSTVVNICRKLSSESPSPFMDAVPILCNLLLYEDR
QLVESVAICLTKITDQVSESPEMLDQLCSHGLIHQSIHLLNLNGRTTLSQ
PVYNGVIGLLRKLSSGSTLAFRTLYELNIGYRLKEIISTYDISHSVSSTN
PITACSNQECNDSDFNVGDENQFTTQVHEVLKLVIGLLPSSPVEDNQLAL
EKESFLVNQPDLLQQFGADVLPVMIQVLKSGANVYVSYGCLSAIHKLTCL
SKSADFVDLLKNANILSVLAGILSRKDHHVVVVALQIAEVLLGKYSEDFL
NSFVKEGVYFAIEALLNSGQQSQGSTDVSEEPVPKETVKCLCQSFERSIS
SSSQTCKIEKDSVYILATRIKESFFGPEVFDSQKGLTDVLQNLKHLSAAL
DDLMTVPIDAPALHDEKFFSVWSQIMERLNGRESVSTFEFTESGVVKALA
NYLSNGLHQRKFSKGDPECDSLPFVGNRFEVFTRSLWSDGEATSSVLIRK
LQNSLSSLESYPIVLSQFLKQRNSFATVPNGRSTSYPILRVRFVKAEGET
CLRDYSQDLVTVDPLCFLDAVDQYMWPKVQLEPLDSVEEKDQAMECPSSQ
LQSTSISCQGESSTHMEIDSPNASQLQGSQEEDQEQLPDSGEENTSSSEE
EDALPRLLFRLEGLELDRSLTVYQAILLHKLKSASETTNDSKLSGSHTIT
YERAPQLAESDENLFPLGSMDNDEYHPFLSFLFAQRLGLRHKATNPPAYD
ILFLLKSLEGMNRFLFHLICHERINAFGEGRLENLDDLRVQVCPVPYAEF
VSSKLTEKLEQQLRDSFAVSTCGLPPWFNDLMGSCPFLFSFEVKTKYFRL
SAFGSQKVHHHPQHLSSEGRPVTGSLPRKKFLACRENILESASKMMELHG
NQKVVIEVEYSEEVGTGLGPTLEFYTLVSRAFQNPDLGMWRCDRSSFAGK
PKEDSGFLVAPSGLFPRPWSDASAAFPDVLQKFVLLGTVVAKALHDGRVL
DLPFSKAFYKLILGQELSSFDIHFVDPELCKTLVELQALARRKKVFSESQ
SDARAAKCDLSFRGTKIEDLCLEFALPGYSDYDLAPHSSTDMVNLDNLEE
YIKAIVNATVCTGIQKQVEAFRSGFNQVFPIEHLGIFNEEELETLLCGER
DLFNMNEVLDHVKFDHGYTSSSPPVENLLEILHEFDKDQQRAFLQFVTGC
PRLPPGGLASLNPKLTIVRKHGSDSSETDLPSVMTCANYLKLPPFSSKVI
SLSSSFVNPLSFLLMLTKAGLRKDLTVLWFCFVFCLVCVLQEKMKEKLIY
AITEGQGSFHLS

>Bol016067/8

METRSRKRAEATSTAPSSSSSSPPPPPSGPTTRSKRARLSSPSSSSAAAATTATAPSSST

RSRSSRSAATATATAAVTPMDTSTESSGFRRGGGRGNRGNDNTNSDKGKEKEHEVRIRDR
ERDRARQQLNMDAAAAAAAAEEDDDNDSEDGNGGFMHPNMSSASSALQGLLRKLGAGLDD
LLPSSGIGSGSSSHLNGRMKKILAGLRSEGEEGKQVEALTQLCEMLSIGTEDSLSTFSVD
SFVPVLVGLLNHESNPDIMLLAARALTHLCDVLPSSCAAVVHYGAVSCFVARLLTIEYMD
LAEQSLQALKKISQEHPTACLRAGALMAVLSYLDFFSTGVQRVAVSTAANMCKKLPSDAS
DYVMEAVPVLTNLLQYHDAKVLEYASICLTRIAEAFASSPDKLDELCNHGLVTQAATLIS
ASNSGGGQASLGVSTYTGLIRLLSTCASGSPLGCRTLLLLGISSILKDILSGSGVSANAS
ISPALSRPADQIFEIVNLANELLPPLPEGSISLPTSANALVKGSGQKNSSPSTSGKQEDS
PKVSPREKLLCDQPELLQQFGLDLLPVLVQIYGSSVNGTIRHKCLSVIAKLMYFSTPEMI
QSLIGDTNISSFLASVLAWKDPQVLVPALQVAEILMEKLPETFSKVFVREGVVHAVDQLV
LVGKPSANASTDQENDCVPGSARSRRYRRRSSNANSDGNQSEELKNSVSASIGATHNSME
SPTASFMLRETVSSCAKAFKDKHFPSDGGEFDVGVTDDLLHLKNLCTKLTAGTNDHKVKG
KGKSKVSGPCLGDFSASKEEYLIGIISEILGELSKGDGVSTFEFIGSGVVAALLNYFSYG
YFSKEKISEVDLPKLRQDGLRRFKAFLEIALPSDGNEGKIPPMTVLIQKLQDALSSLERF
PVVLSHPSRSLSGSARLSSGLSALAHPLKLRLCRAPGEKALRDYSSNIVLIDPLASIAAV
EEFLWPRVQRSESGVKAAAPAGNTEPGTLPSGAGVSSPSSSTPASTTRHSSRSRSAIKIG
DASKKEPVHEKGTSSSKGKGVMKPAQPDKGPQTRSSAQRKAVLDKDTLMKPASGDSSSED
EEMDISPVDMDDALVIEEEDISDDDDDDDEEDVLDDSLPMCTPDKVHDVKLGDAVDDEGA
GLAPSGRQMNSALAGSSGTATARGSNSTDAGIGNLYGSRGALSFAAAAMAGLGAASGRGI
RGSRDLHGRTLNRSSDESSKLMFTAGGKQLSRHMTIYQAVQRQLMLDEDDDDRLGGSDFI
SSDGSRLNDIYTIMYQMPDSQANRLSAGGASSTTPSKSTKSATTNASVEAQSYRASLLDS
IVQGKLPCDLEKANSTYNVLALLRVLEGLNQLGPRLRAQTISDRFAEGKITSLDDLNTTA
AKVSHEEFINSKLTPKLARQIQDALALCSGSLPSWCYQLTTACPFLFPFQTRRQYFYSTA
FGLSRALNRLQQQQGADGSGSTNEREMRIGRLQRQKVRVSRNRILDSAAKVMEMYSSQKA
VLEVEYFGEVGTGLGPTLEFYTLLSHDLQKVSLGMWRSNSGDKLSMQTDRDEIQDGKSAA
ARDRDIVQAPLGLFPRPWPSTADVSEGSRFHKVVEYFRLLGRVMAKALQDGRLMDVPLST
AFYKLILGQELDLHDVIIFDTELGKTLQELRVLVGRKHYLEAEGGDNSSVISDLCLRGSR
IEDLCLDFTLPGYPEYILRPGDDIVDINSLEDYISLVVDATVKRGVARQIEAFRSGFNQV
FDIKSLQVFTPSELDYLLCGRRELWEAETLVEHIKFDHGYTAKSPAIIFLLEIMGELTAD
QQRAFCQFVTGAPRLPPGGLAVLNPKLTIVRKLSSTSNAAANGTGASETADDDLPSVMTC
ANYLKLPPYSTKEIMYKKLLYAINEGQGSFDLS
>Bol030543
MTLSRYSDNHKRKFDDNGVDNNILHKMKRHEVDADHTSPSQQPFNPWRSD
ENSPQNRYECSSSSSSSSSSSSSTESSTRSESTRLQLFVRMVSGGKTIVI
QADKSDTVENLHQRIELKTKIPVNGGKTIVIQADKSDTAENLHQRIELKT
KTPVNKQRVIYKGKQLQDERPLSYYSIPQDSSLHLVGRMRSTDHPVACRV
VDDIMYIVSRMHRGGGGGENNNLPRGMSINEKLSSFFSAIPRESGGDDNS
YIDSTDKYLTIFTNSSVPASLVMLYTSPLKANKSCGESSIKLFLNSCLAL
PDEQQSCCLPVVLEFCRLLRRVCPHSSLYGSCRNTLGSLLETVQELVDKS
GTEVTSLHYRLLTVGEEILPCLSELADLMEQQLYDDNLGPSLYDVQKFSS
FWRPLRYAIDFQLSSLIPFALPLRSTVLEAEVARICQIFGRLLTTMHICI
LRIESSLGGRGVANTEAMPLKWGQYLNILKIVSSMSELYQGGKEKVVSLI
NSRKVPFCALILKFVKRGDDHGWISEYREATTFECRRHLAMMLFPDGKED
YSEMHEMLIDRSQVLKESYEYISQASPGGLHGALFMEFKNEEATGPGVLR
EWFYLVCQEIFSPGGTLFLRSPDDFRRFSPNPASKVEQLHLGYFKFAGRV
IALALMHKVQVGVLFDRVFYLQLTNQTISLEDIKDTDRVIYNSCKQILEM
DPVFFDSNAGLGLNFVLETEELGKRETKELIKDGKSTAVDSKNREEYVKL
LISERFVTSVSELVEKFSEGFSDILSVPIQSFFRHLDLEDFDGMLRGGEN
QISVDDWKAHTEYNGFKETDRQIDWFWKILRKMTEEERRSVLFFWTSNKF
IPLEGFRGLSSKLYIYRLHEANDRLPTSHTCFYRLCLPKYPTMGLMEQRL
RFITQDHVSSSFGKW
>Bol036879
MFFSGDPSTRKRVDLGGRSTKERDARKLLEQTRMERNRRLLQKQQNAAAL
KIQKFFRGRRSVATERSKVRREFCETYGDNCQNVDRHCFEPGSSFLRQFL
FFFKAKNSGDFLILVETCRLLKMFAHSNGDILSLFSGSDYSAEHNLVDFR
VKELAFTCIEAIHQNRDRLRDQLLVTTEEASTSTAILMEAMSLVLDPKLP
WVCKTVSYLQKRHIFKLVREIVITAKENPRGQTKTGNILSLERVLILVVS
HVGREPCCCPVDDPRWSFSSMILTLPLIWQLFPNLKVVFANPNLSQHYIH
QMAFCIQKDTHVLPIDTSTEFPGYACLLGNTLETANVVLSQPDCSLDMAV

DIASVATFLLEKLPPVKSSERESKQSSSEEDDMLIDDIPELVLNKTLEQQ
ITSNAIDSRFLLQLTNVLFHQVSLGTQPYDEDKEAQAIGTASSFLYAAFN
TLPLERIMTVLAYRTELVVVLWNYMKRCHENQKWSSMPNLLAYLPGDAPG
WLLPLVVFCPVYKHMLMIVDNEEFYEREKPLSLQDIRLLIIILKQALWQL
LWVNPLTQPSTGKSVSNNLSKKNPVGLIQNRVGVVVAELLSQLQDWNNRQ
QFTSSSDFQADTVNEYFISQAIAEGTRANYILMHAPFLIPFTSRVKIFTT
QLATARESHGHGSQAIFARNRFRIRRDHILEDAYNQMSALSEDDLRGSIR
VTFVNELGVEEAGIDGGGIFKDFMEKITRAAFDVQYGLFKETVDHMLYPN
PGSGMVHDQHLQFFHFLGTLLAKAMFEGILVDIPFATFFLSKLKHKYNYL
NDLPSLDPELYKHLIFLKRYKGNIAELELYFVILNNEYGERTEEELLQGG
KDMRVTNENVITFIHLVSNHRLNFQIRQQSSHFLRGFQQLIPKECIDMFN
EHELQVLISGSADYLDIDDLRQNTNYTGGYNAGHYVIDMFWEVLKSFSTE
NQKKFLKFVTGCSRGPLLGFKYLEPAFCIQRAAGSASNEAVDRLPTSATC
MNLLKLPPYQSKEQLETKLMYAISAEAGFDLS

>Bol003946

MTRRLTSSLSHRSSSSDNPKRKLDENGVENLLLPKMKRHEVSAESSSGSESTRLQIFVRM

MSGGKTTVIHADKNDTVEQLHHRIELKTKIPAKEQRVIYKGKQLQLEHSLSYYSIEQDSS

LHLLGRMQSTEHPVAWRTVDDIMYAVSRMLRGDWFVSVNNINDILVTFFDTMPKKATCEY

LEIFLNSSVPAALVMLFASPLETNKSCAKSSIKLFFDSCIDLPDHEQRCFLPVVLEFFRL

LRKVCPRNKLYVSCRNTLGSVLESVYSSGESRSCLLSIGDEIFPCVRELAKLMVKQLVDD

DDDSGRPSSCDVQKVSSFWLPLRYSIQNQAETFTLIAFPLQNNVHGKLYHIFEKLLNAMG

EGMSRLESILARRGAVSAKCSHYLAILKIVSSMSELYQGGKERVARLLSSRKASFGALVL

KFAKRGDDDDHQWIFEYKEATTFESRRHLAMLLFPDVEEEEEDYEEMHEMLIDRSNLLAE

SFEYITEASSEELHGRLFMEFKNEEATGPGVLREWFYLLCQEIFNPQNTLFTRSADDSRR

FSPNPASEVDPLHLDYFEFTGRVIALALMHKVQVGVLFDRVFFLQLTGKKIGLEDIKNTD

RIMYNSCKQILEMDPECFDSDAGLGLTFVSETEVLGKRETKELLKDGKSIAVNSKNREQY

VNLLIKHRFATSVSEQVNQFSQGFSDILADTACFFERLYVEDFDCMLGGGDNLISIDDWK

AHTEYNRFKKTDRLIGWFWKILKKMTEGERRSVLFFWTSTRFIPVEGFRGLSSKLYIYRL

HEAKDHLPTSHTCFYRLCLPKYPKIGLMEKRLRLIAQDHVSSSFGQW

>Bol000770
MENRGQKRMEVLGEPPADKRACNSQDFTSGASSVQAQQANGNTDADMDTS
SSASPSSRSGGEQDREEKEEEDYGSCDSDDADPRRRVLQRYQRGRSTGDQ
LKLKSLVTRLSEENDPSLQLTGLTELCEVLSFCTEDSLSSVMADLLSRVL
VKLAKHESNADIMLLAIRGITYLCDVYPRSVAFLVKHETVPALCQRLLTI
EYLDVAEQCLQALEKISRDQPVACLNAGAIMAVLSYIDFFSTSIQRVAVS
TVVNICRKLPSEPPSPVMDAVPVLCNLLQYEDRQLVESVAICLTKIADQV
SQSPTMLDQLCSHGLIHQSTHLLNLNSRTTLSQPVYNGVIGLLRKLSSGS
TLAFRTLYELNIGYRLKEIISTYDISHSVSSTQPLNACSNQVHEVLKLVI
ELLPASPVEENHLALEKESFLVDQPNLLQQFGADMLPVMTQVLKSGASVY
VSYGCLSAIHKLTCLSKSGDLVELLNNANISSVLAGILSRKDHHVVVVAL
QIAEVLLEKYRDAFLNSFIKEGVFFAIAALLTADRGQQINPVSGFIQGSV
PKEIVKCLCQSFEGSVSSSSQTCKVGNDSVYILATRIKESFFGPEVFDSQ
KGLTDVLQNLKNLSAELNDLVTVPVDTHVLHGERFFSIWNQIMERLNERE
SVSTFEFTESGVVKAVANYLSNGLYERKLSKGGPECDSLPLVGNRFEVFT
RLLWSDGEATPSVLIQKLQNSLSSLENYPIVLSQLLKQRNCYAAVPNGRC
ISYPVLRVRFAKAEGETCLRDYSPNFVTVDPLCYLDAVGQCLWPQVNLKP
LNSVEAKDQAIECQSSQLQSTSISCQGESSSHMEIDSPNASQLQGSQEEE
DQDHLIDSGEENSSSSKEEDVRPRLLFRLEGLELDPSLTVYQAILSHKLK
SENETTNDSKLSGHHTITYERAPQLAVSRENLFPLGSIDNDEHHPFLSYL
FAHRLGLRHRVTSPPAYAILFLLKSLEGMNRFLFHLICHERINAFGEGRL
ESLDDLSVQVRPVPYAEFVSSKLTEKLEQQLRDSFAVSTCGLPPWFNDLM
YSCPFLFSFEVKSKYFRLAAFGPQQVHNQPQHLGSSNVHGEGRSVTVSLP
RKKFLACREKILESAAKMMELHGTQKVSVEVEYSEEVGTGLGPTLEFYTL
VSRAFQNPDLGMWRSDPSSLAGKPMVPPSGLFPRPWSATSAAFPGVLQKF
VLLGTVVAKALQDGRVLDIPFSKTFYKLILGQELSSFDIHFVDPELCKTL
VELQALARRRKVISESQSDVRAAKSDLSFRGTKIEDLCLEFALPGYTDYV
LSPQFANDMVNLGNLEEYVKAIVNATVCNGIQTQVAAFRSGFNKVFPIEH
LKIFNEEELETLLCGERDLFSMNEVLDHIKFDHGYTSSSPPVQNLLEILH
EFDKEQQRAFLQFVTGCPRLPPGGLASLSPKLTIVRKHGSDSSETDLPSV
MTCANYLKLPPYSSKEKMKEKLIYAITEGQGSFHLS
>Bol003376
MATGRSASMPSRLRQLLSGEGSIGPSIRLDAEPPPEIKSFIEKVIQSPLS
DIAIPLSGFRWEYSKGNFHHWRPLFLHFDKYFKTFISTRNDLLLSDHILE
DADPFPKYSVLQILRVMQIILENCPNKSSFDGLEHFKLLLASTDPEVLIA
ALETLSALVKISSSKLHRSGKLIGCGSVNSFLLSIAQGWGSKEEGLGLYS
CVVANERNQEEGLSIFPSDLENNHKEADFRIGSTVYFELRGHSAQRIDDG
SSSATSSSSSSSSRVIQIPDLHLRKEDDLVLMKECIEHYNVPPELRFSLL
TRVRYAHAFRSSRICRSYSRICLLAFIVLVQSSDAQEELASFFANEPEYT
NELIRIVRSEEPIPGTIRTLAMLALGAQLAVYSASHDRARILSRSSISFA
VGNRMILLNVLQKAVLSLKISSDPSSIAFVEALLQFYLLHIVSSSSSGST

IRGSGMVPTFLPLLEYTDPSHLHLVYLAVKALQKLMDYSSSAVPLLRDLG
GVELLSQRLELEVHHILKLTGENNSDMVVGESLELNGDQLLSRKRLIKVL
LKSLGSSTYTPGNANRSQSSQESTLPATLSLIYRNADKFGGDIYYSAVTV
MSDLIHKDPTSLTSLFEMGLPEAFLSSVVSGVLPSSKAIACIPNGLGAIS
LNSKGLETVKETSALRFLVDVFTSKKYVLAMNEAIVPFTNAVEELLRHVS
SLRATGVETIIEIVDNIACFGESGSSSSSVSTAMEMDSDVKNINGVSHEQ
FVQLCIFHLIILLHRTMENAETSRLFVEKSGIEALLKLLLRPSIAQSSEG
VSIALHSTIVFKGFTQHHSASLARAFCSSLRDQVKKALGGLQDLSGTFLL
DRKLSPSCGIFSSLFLVEFLLFLAASKDNRWMTALLSEFANGSKDVLENI
GHLHREILWHIAVYESGTLESPNTSPESEQTEPSSNGTEGQRFSSFRQFL
DPLFQRRTSGWSAESQFFDLTNLYRDLGRASTGFQPRPISDGPSSSSEAS
GNRELEREGSYYTSCCDMVKSLSFHITYIFQELGKAMLLQFRRREDTVNV
SPSSKLVASSFASISLDHLNFEGHEIAAEASRSTKCLYLGKVVDFIDAVL
LDRPDSCNPVLINCFYGRGVIQTVLTTFDATSQLLFSINRSYSSPMETDD
VKGKQDEKEDTDHAWIYGPLAGYGKLMDHLVTSSFILSPFTKRLLVQPVE
NGNIPFPQDAETFVKILQSIVLKAVLPVWTHPLFTECSYDFVTALISIIK
HVYSGVELKSLGGSGSARVTGPPPSETTISTIVEMGFSRSRAEEALRQVG
SNSVEFAMEWLFSHPEEIQEDDELSRALAMSLGNSESDTKENVVDETREQ
IEAEIVSLPPVEELIATCTKLLQMKEPLAFPVRDLLVLICSENNGEHRSG
GICCLLSRIKDCCPVFDDTKNNLLSALLHVLALILHEDAGSREVALKAGT
VRLVCDVLSKWDSGSIDKEKFNVPKWVTTGFLAIDRLLQVDQKLNTELIE
ELQKGETSLAIDESKQDKLQSVFGSSQLVDADDQKKLIEIACTCIRNQLP
SETMHAVLQLCSTLTRKHSVAVCFLDFGGVQGLLSLPSNSLFPGFDSVAA
SIIRHVLEDPQTLQQVMESEIKHALATLSNRHSNQRISPRNFLLHVNSVI
ARDPVTFIQAARSICQVEMVGERPYIVLVKEKEKSKDKEKDKDKDKDRAD
KEKSQTSNEVATTTPPGSTKAKVYRKPPQSFIGVVELLLDSLCNFVPPPK
DDMVEGDSTSADMDIDHASTKGKGKAVATTPEEKKAISQDMSASLAKIVF
ILKLLSEMLLMYSSSIHIILRRDAEIKSLRGPQQKGGQVGGIFHHILHKF
IPYSRVKKEKKSESDWRQKLASRANQFLVGASVRSAEARKRIFSDISSIF
NDFIGTSNGLRPPANEIHILIDLLSDMLSARSPTGSHISSEASNTFVDVG
LVKSLTRTLEVLDLDNVESPKTVTGIIKVLELVTKEHAHSADSNSKNENA
NKSSDQIQSGRGDTTADASEAGGILLRSNHDSMTADHAENFGGSHTFVGS
EDVTDDMEHDQDLDEGFAAGGDDYMQEAAEDARGLENGIGSMGIEFEMHT
HVQENLDEEDEEDEDDEDRVILRFEDGFNGLNVLDHLEVLRDHRFSDETL
HVMPVEVFGSRRQGRTTSIYSLLGRTGDGATPSQHPLLSGSPSLPASQSQ
TESTHDHTVGGRDSNGSSSSRLDAIFRSLRNGRQGHRLNLWADDSQQIVG
SGASTVPQGLEDLLVSQLRRPGSDNPSDDQNPSPLEPQSQAESGQPQEAT
VRPEIPDENATDNGGANVSAPSIVSPVASATPDTRAPATDSVSSSRSQSV
EMQYDPNDSTVRDVEAVSQESGGSGATLGESLRSLDVEIGSADGHDDGGE
RHDVQPAIRSRRANLSLLPSSTGREASLYSVTEVPENSGHEAEQDNPPEE
QPVNRNVASSSIDPAFLDALPEGLRAEVLSAQQGQAPEPSSNEQQNSGDI
DPEFLAALPADIRAEVLAQQQAQRVHQSHELEGQPVEMDTVSIIATFPSE
LREEVLLTSDDAVLANLTPALVAEANMLRERFAHRYHNRALFGMHPRHRR
GEASRRGEGVISGNEGIASRRSAAKVIEAAGAPLVNTEALQAMIRVLRIV
QPLYKGPLQRLLLNLCSHGETRFSLVNTFMDMLMLDTRKPVNYSSVSEPP
YRLYACQSNVTYSRPQHFDGVPPLVSRRVLETLTYLARNHIYVAKILLQS
RLSLPSLQGSVPSDKARGKAVVVSDDHMSRTQQEPESVAFALLLSLLNQP
LYLRSVAHLEQLLNLLEVIIDNAERKSESADGSDGSASEQQSTHQALEVE
NNSENHDMVSGTVPAGTVTKPIVSSGSSSNRAESECDVHTVLLNLPQSEL
CLLCSLLAREGLSDNAYTLVAEVLKKLVAMAPSHCHLFITELANAIQSLT
RSAISELHMFGEAVKTLLSTTSSDGSGVLRVLQALSSLVDSLLITKEKNS
EEHVAVFSQLSNINLALEPLWLELSNCICKIEGHSDSASVTTTSPTTSTS
SATTRGAGVSQSLPAGAQNMLPYVESFFVTCEKLHPSSQSCDISVPMASS
DAEEQPKGPGPSSSSKVDEKYGSFIKFSERHRKLLNAFIRQNPALLEKSF
SLMLKVPRFIEFDNKRAYFRSKIKHQHDHHHSPLRISVRRAYILEDSYNQ
LRMRSTQELKGRLTVHFQGEEGIDAGGLTREWYQLLSRVIFDKGALLFTT
VGNDSTFQPNPNSVYQTEHLSYFKFVGRVVGKALFDGQLLDVHFTRSFYK
HILGVKVTYHDIEAIDPDYYKALKWMLENDISDVLDLTFSVDADEEKLIL
YEKTEVTDHELIPGGRNIKVTEENKHEYVDLIAEHRLTTAIRPQINAFLE
GFSELILKDLISIFNDKELELLISGLPDIDLDNLRANTEYSGYSPGSPVI
QWFWEVVQGLSKEDKARLLQFVTGTSKVPLEGFSSLQGISGAQKFQIHKA
YGSANHLPSAHTCFNQLDLPEYPSKEHLQERLLLAIHEASEGFGFG
>Bol000411*
QPAIRSRRANLSLVPSSTGREASLYSVTEVPENSGHEAEQDNPPEEQPVNRDVASSSIDP
AFLDALPEGLRAEVLSAQQGQAPEPSSNEQQNSGDIDPEFLAALPADIRAEVLAQQQAQR
VHQSHELEGQPVEMDTVSIIATFPSELREEVLLTSDDAVLANLTPALVAEANMLRERFAH
RYHNRALFGMHPRHRRGEASRRGEGVISGNEGIASRRSAAKVIEAAGAPLVNTEALQAMI
RVLRIVQPLYKGPLQRLLLNLCSHGETRFSLVNTFMDMLMLDTRKPVNYSSVSEPPYRLY
ACQSNVTYSRPQHFDGVPPLVSRRVLETLTYLARNHIYVAKILLQSRLSLPSLQGSVPSD
KARGKAVVVSDDHMSRTQQEPESVAFALLLSLLNQPLYLRSVAHLEQLLNLLEVIIDNAE
RKSESADGSDGSASEQQSTHQALEVENNSENHDMVSGTVPAGTVTKPIVSSGSSSNRAES
ECDVHTVLLNLPQSELCLLCSLLAREGLSDNAYTLVAEVLKKLVAMAPSHCHLFITELAN
AIQSLTRSAISELHMFGEAVKTLLSTTSSDGSGVLRVLQALSSLVDSLLITKEKNSEEHV
AVFSQLSNINLALEPLWLELSNCICKIEGHSDSASVTTTSPTTSTSSATTRGAGVSQSLP
AGAQNMLPYVESFFVTCEKLHPSSQSCDISVPMASSDAEEQPKGPGPSSSSKVDEKYGSF
IKFSERHRKLLNAFIRQNPALLEKSFSLMLKVPRFIEFDNKRAYFRSKIKHQHDHHHSPL
RISVRRAYILEDSYNQLRMRSTQELKGRLTVHFQGEEGIDAGGLTREWYQLLSRVIFDKG
ALLFTTVGNDSTFQPNPNSVYQTEHLSYFKFVGRVVGKALFDGQLLDVHFTRSFYKHILG
VKVTYHDIEAIDPDYYKALKWMLENDISDVLDLTFSVDADEEKLILYEKTEVTDHELIPG
GRNIKVTEENKHEYVDLIAEHRLTTAIRPQINAFLEGFSELILKDLISIFNDKELELLIS
GLPDIDLDNLRANTEYSGYSPGSPVIQWFWEVVQGLSKEDKARLLQFVTGTSKVPLEGFS

SLQGISGAQKFQIHKAYGSANHLPSAHTCFNQLDLPEYPSKEHLQERLLLAIHEASEGFGFG
>Bol000412*
QPAIRSRRANLSLLPSSTGREASLYSVTEVPENSGHEAEQDNPPEEQPVNRDVASRSIDP
AFLDALPEGLRAEVLSAQQGQAPEPSSNEQQNSGDIDPEFLAALPADIRAEVLAQQQAQR
VHQSHELEGQPVEMDTVSIIATFPSELREEVLLTSDDAVLANLTPALVAEANMLRERFAH
RYHNRALFGMHPRHRRGEASRRGEGVISGNEGIASRRSAAKVIEAAGAPLVNTEALQAMI
RVLRIVQPLYKGPLQRLLLNLCSHGETRFSLVNTFMDMLMLDTRKPVNYSSVSEPPYRLY
ACQSNVTYSRPQHFDGVPPLVSRRVLETLTYLARNHIYVAKILLQSRLSLPSLQGSVPSD
KARGKAVVVSDDHMSRTQQEPESVAFALLLSLLNQPLYLRSVAHLEQLLNLLEVIIDNAE
RKSESADGSDGSASEQQSTHQALEVENNSENHDMVSGTVPAGTVTKPIVSSGSSSNRAES
ECDVHTVLLNLPQSELCLLCSLLAREGLSDNAYTLVAEVLKKLVAIAPSHCHLFITELAN
AIQSLTRSAISELHMFGEAVKTLLSTTSSDGSGVLRVLQALSSLVDSLLITKEKNSEEHV
AVFSQLSNINLALEPLWLELSNCICKIEGHSDSASVTTTSPTTSTSSATTRGAGVSQSLP
AGAQNMLPYVESFFVTCEKLHPSSQSCDISVPMASSDAEEQPKGPGPSSSSKVDEKYGSF
IKFSERHRKLLNAFIRQNPALLEKSFSLMLKVPRFIEFDNKRAYFRSKIKHQHDHHHSPL
RISVRRAYILEDSYNQLRMRSTQELKGRLTVHFQGEEGIDAGGLTREWYQLLSRVIFDKG
ALLFTTVGNDSTFQPNPNSVYQTEHLSYFKFVGRVVGKALFDGQLLDVHFTRSFYKHILG
VKVTYHDIEAIDPDYYKALKWMLENDISDVLDLTFSVDADEEKLILYEKTEVTDHELIPG
GRNIKVTEENKHEYVDLIAEHRLTTAIRPQINAFLEGFSELILKDLISIFNDKELELLIS
GLPDIDLDNLRANTEYSGYSPGSPVIQWFWEVVQGLSKEDKARLLQFVTGTSKWETDVLN
STTQGTSGRI
>AT4G12570/UPL5
MTLSRSSADDSTNNANRSYSAVAGTDNKRKRDEDSSDYVGVAESLEMLKK
QEIDADHMAASAQQTLISWRSGENSRSLSSSGECSSSNRPESTRLQIFVR
MMSGGKTIVIHAEKYDTVEKLHQRIEWKTKIPALEQRVIYKGKQLQRENS
LTYYSIEQDASLQLVARMQSTEHPVAWQTIDDIMYTISRMYKGENLQSNI
NEKIVTFFAMIPVESDESIAKYLNIFSNSSVPAALVMLYASSLERNKSCA
KSSVKLFLSNCVALPKNQKNYCLPIVLEFCKLLRKVCPDQKLYVTCRNTL
GSMLETFDNPHGVYNDQYETFGVEIFPFFTELTGLLLNELAQNSGPSFCD
FQKVSSFWQQLRKVIELKVAFPIPIVLPMQSTALEAEIRHLHRLFGSLLT
TMDLCMCRVESSLADKEVGNSETMSSSWSQYLSILKIINSMSNIYQGAKG
QLAVMLNKNKVSFSALVVKFAKRGDDHQWIFEYKEATNFEARRHLAMLLF
PDVKEDFEEMHEMLIDRSNLLSESFEYIVGASPEALHGGLFMEFKNEEAT
GPGVLREWFYLVCQEIFNPKNTLFLRSADDFRRFSPNPASKVDPLHPDFF
EFTGRVIALALMHKVQVGVLFDRVFFLQLAGLKISLEDIKDTDRIMYNSC
KQILEMDPEFFDSNAGLGLTFVLETEELGKRDTIELCPDGKLKAVNSKNR
KQYVDLLIERRFATPILEQVKQFSRGFTDMLSHSVPPRSFFKRLYLEDLD
GMLRGGENPISIDDWKAHTEYNGFKETDRQIDWFWKILKKMTEEEQRSIL
FFWTSNKFVPVEGFRGLSSKLYIYRLYEANDRLPLSHTCFYRLCIPRYPT
ITLMEQRLRLIAQDHVSSSFGKW
>AT5G02880/UPL4
MENRGQKRMEVVEELPADKRACNSQDFRPSTSGSSVQAQANDTNPGHENV
DADMDTSSSASPSSRSDEEEQEEQDKEDSDYGSCDSDEEDPRQRVLQDYQ
RQRSSGDHGKLKSLLLNLTGETDPSGQLSRLTELCEVLSFSTEESLSSVM
ANMLSPVLVKLAKHENNADIMLLAIRAITYLCDVYPPSVEFLVRHDTIPA
LCQRLLTIEYLDVAEQCLQALEKISRDEPVACLNAGAIMAVLSFIDFFST
SIQRVAISTVVNICKQLSSESPSPFMDAVPILCTLLQYEDRQLVENVAIC
LTKIADQASESPAMLDQLCRHGLINESTHLLNLNSRTTLSQPVYNGVIGM
LRKLSSGSALAFRTLYELNIGYSLKEIMSTYDISHSVSSTHPINACSNQV
HEVLKLVIELLPASPVEDNQLASEKESFLVNQPDLLQQFGRDMLPVMIQV
LNSGANVYVSYGCLSAIHKLTCLSKSGDIVELLKNTNMSSVLAGILSRKD
HHVIVVALQVAEVLLEKYRDTFLNSFIKEGVFFAIEALLSSDRGQQNQGS
ADLSQKPVTKEIVKCLCQSFERSLSSSSQTCKIEKDSVYVLATRIKEGFF
GPEVFNSEKGLTDVLQNLKNLSVALSELMTVPIDAHVLHDEKFFSIWNQI
MERLNGRESVSTFEFIESGVVKSLASYLSNGLYQRKLSKGGPECDSLPFI
GKRFEVFTRLLWSDGEATSSLLIQKLQNSLSSLENFPIVLSQFLKQKNSF
AAIPNGRCTSYPCLKVRFLKAEGETSLRDYSQDFVTVDPLCYLDAVDQYL
WPKVNIEPIDSVEAKDQAIECQSSQLQSTSISCQAESSSPMEIDSESSDA
SQLQGSQVEDQTQLPGQQNASSSETSSEKEDAVPRLLFRLEGLELDRSLT
VYQAILLHKLKSESEATNDSKLSGPHNITYERSAQLGDSRENLFPPGSME
DDEYRPFLSYLFTHRLALRLKGSSHPPYDILFLLKSLEGMNRFLFHLISL
ERINAFGEGRLENLDDLRVQVRPVPHSEFVSSKLTEKLEQQLRDSFAVST
CGLPPWFNDLMDSCPCLFSFEAKSKYFRLAAFGSQKIRHHPQHLSSSNVH
GEARPVTGSLPRKKFLACRENILESAAKMMELYGNQKVVIEVEYSEEVGT
GLGPTLEFYTLVSRAFQNPDLGMWRNDCSFIVGKPVEHSGVLASSSGLFP
RPWSGTSTTSDVLQKFVLLGTVVAKALQDGRVLDLPLSKAFYKLILGQEL
SSFDIHFVDPELCKTLVELQALVRRKKLFAEAHGDSGAAKCDLSFHGTKI
EDLCLEFALPGYTDYDLAPYSANDMVNLDNLEEYIKGIVNATVCNGIQKQ
VEAFRSGFNQVFSIEHLRIFNEEELETMLCGECDLFSMNEVLDHIKFDHG
YTSSSPPVEYLLQILHEFDREQQRAFLQFVTGSPRLPHGGLASLSPKLTI
VRKHGSDSSDTDLPSVMTCANYLKLPPYSSKEKMKEKLIYAITEGQGSFH
LS
>AT4G38600/UPL3
METRSRKRAEATSAAPSSSSSSPPPPPSASGPTTRSKRARLSSSSSSSLA
PTPPSSSTTTRSRSSRSAAAAAPMDTSTDSSGFRRGGRGNRGNNNDNSDK
GKEKEHDVRIRERERERDRAREQLNMDAAAAAARSADEDDDNDSEDGNGG
FMHPNMSSASSALQGLLRKLGAGLDDLLPSSGIGSASSSHLNGRMKKILS
GLRAEGEEGKQVEALTQLCEMLSIGTEDSLSTFSVDSFVPVLVGLLNHES
NPDIMLLAARALTHLCDVLPSSCAAVVHYGAVSCLVARLLTIEYMDLAEQ
SLQALKKISQEHPTACLRAGALMAVLSYLDFFSTGVQRVALSTAAN MCKK
LPSDASDYVMEAVPLLTNLLQYHDSKVLEYASICLTRIAEAFAPYPEKLD

ELCNHGLVTQAASLISTSNSGGGQASLSVSTYTGLIRLLSTCASGSPLGF
RTLLLLGISSILKDILLGSGVSANASVSPALSRPADQIYEIVNLANELLP
PLPEGVISLPTSTNALVKGSCQKKSSPSTSGKQEDILKISPREKLLGDQP
ELLQQFGLDLLPVLVQIYGSSVNGTIRHKCLSVIGKLMYFSSSEMIQSLI
GDTNISSFLAGVLAWKDPQVLVPALQVAEILMEKLPETFSKVFVREGVVH
AVDQLVLVGKPSHASPTDKDNDCVPGSARSRRYRRRSSNANSDGNQSEEP
KNPASLTIGANHNSLDTPTASFMLRETVSSCAKAFKDKYFPSDGGDVDVG
VTDDLLHLKNLCTKLTAGIDDHKVKGKGKSKASGPFLGDFSASKEEYLIG
VISEILGEISKGDGVSTFEFIGSGVVAALLNYFSCGYFSKEKISELNLPK
LRQEGLRRFKAFLEVALPFDGNEGKVPPMTVLIQKLQNALSSLERFPVVL
SHPSRSLSGSARLSSGLSALAHPLKLRLCRASGEKTLRDYSSNIVLIDPL
ASLAAVEEFLWPRVQRSESALKPAAPIGNTEPGTLPSGAGVSSPSSSTPA
STTRRHSSRSRSAINIGDTSKKDPVHEKGTSSSKGKGKGVMKPAQADKGP
QTRSNAQKRAVLDKDTQMKPASGDSSSEDEELEISPVDIDDALVIEEDDI
SDDEDDDNEDVLDDSLPMCTPDKVHDVKLADSVDDDGLATSGRQMNPASG
GTSGAAAARASDSIDTGIGNSYGSRGALSFAAAAMAGLGAASGRGIRGSR
DLHGRTLNRSSDEPSKLIFTAAGKQLSRHLTIYQAVQRQLMLDEDDDDRF
GGSDLVSSDGSRFNDIYTIMYQRPDSQVNRLSVGGASSTTPSKSTKSATT
NSSVESQSHRASLLDSILQGELPCDLEKSNSTYNVLALLRVLEGLNQLCP
RLRAQTLSDRFAEGKITSLDDLSTTAAKVPLDEFVNSKLTPKLARQIQDA
LALCSGSLPSWCYQLTRACPFLFPFQTRRQYFYSTAFGLSRALNRLQQQQ
GADGSGSTNEREMRIGRLQRQKVRVSRNRILDSAAKVMEMYSSQKAVLEV
EYFGEVGTGLGPTLEFYTLLSHDLQKASLGMWRSSSGDKVSMQIGRDEIE
DGKPSAANRDIVLAPLGLFPRPWPSTADISEGGQFHKVIEYFRLLGRVMA
KALQDGRLLDVPLSTAFYKLILGQELDLHDIVLFDAELGKTLQELRVVVA
RKHYLEGVGGDNSSTISDLCLRGCRIEDLSLEFTLPGYPEYILRSGDEIV
DITNLEEYISLVVDATVKRGVTRQIEAFRSGFNQVFDITSLQIFTPSELD
YLLCGRRELWEVETLAEHIKFDHGYNAKSPAIINLLEIMGELTADQQRAF
CQFVTGAPRLPPGGLAVLNPKLTIVRKHSSTSSAAANGAGASETADDDLP
SVMTCANYLKLPPYSTKEIMYKKLLYAINEGQGSFDLS
>AT3G17205/UPL6
MFFSGDPSTRKRVDLGGRSTKERDARKLLEQTRMERNRRLLQKQQNSAAL
KIQKFFRGRRSMAIERSKVRHDFCETYGNNCQNVDRHCFEPGSSFLRQFL
FFFKAQNSGDFVILVETCRLLQSFVDSSGDIVSLFSGLDYSSEHNLVDFR
VKKLAFTCIEAIHQNRNRLRDQLLVTPEEASISTAILMEAVSLLLDPKLP
WVCKIVSYLQKRKVFKLVREMVTTAKESPRGKTMTGNILSLERVLILIVP
HIGREPCCCTVVDPRWSFSSMILTIPLIWKLFPNLKVVFANPSLSQHYIH
QMASCIQKDTCVLPMETSPEFPGYACLLGNTLDTANVVLSQPECSLDMAI
DIALVATFFLETLPPVKSSEGESRQGSSDEDDMLIDDVPGLVLNKALEQQ
ITNAIDSRFLLQLTNVLFRQVSLGMQSYDEDKEALAIGTASSFLYAAFNT
LPLERIMTILAYRTELVAVLWNYMKRCHENQKWSSMPKLLAYLPGDAPGW
LLPLVVFCPVYKHMLMIVDNEEFYEREKPLSLQDIRLLIIILKQALWQLL
WVNPLTQPNTGKSVSNDLSKKNPIELIQNRMGIVVSELLSQLQDWNNRKQ
FTSSSDFQADSVNEYFISQAIMEGTRANYILMQAPFLIPFTSRVKIFTTQ
LATARQSHGSQGIFARNRFRIRRDHILEDAYNQMSALSEDDLRSSIRVTF
VNELGVEEAGIDGGGIFKDFMEKITRAAFDVQYGLFKETADHMLYPNPGS
GMIHEQHLQFFHFLGSLLAKAMFEGILVDIPFATFFLSKLKQKYNYLNDL
PSLDPELYRHLIFLKRYKGDISDLELYFVILNNEYGERTEEELLPGGQDM
RVTNENVITFIHLVSNHRLNFQIRQQSSHFLRGFQQLIPKEWIDMFNEHE
LQVLISGSVDSLDIDDLRNNTNYAGGYHAGHYVIDMFWEVMKSFSTENQK
KFLKFVTGCSRGPLLGFKYLEPAFCIQRAAGSASNESVDRLPTSATCMNL
LKLPPYQSKELLETKLMYAISAEAGFDLS
>AT1G55860/UPL1
MAIAEEVTVFAETSLGTRIVMAVPLDITAADFKRKLEKTHASCLSSVGEI
EVHGLMVKRKSRFYHLVESVPIRYIFLDNQKPWFIHAEARVVSRSQESSV
SNSIDKTHIRHCHGSDNSIERVTALIQGKDKKIKKSRAKSDSRNVASGQE
TCLDKREYLGPSTSEPETIKSLPSIARVAEMKNSECFKSFTPKPPERKPV
DSESESIGKRITVAANNIRMQGKTHNSSSLSSSIFRSNSCRNRSLDGKII
TSLAKFMVFEPSKIKSFINSVTSVPLELIHEPLACFRWEFDKGDFHHWVD
LFNYFDTFFEKHVQVRKDLHIEENFEESDPPFPKDAVLQVLRVIRVVLEN
CTNKHFYSSYEQHLSLLLASTDADVVEACLQTLAAFLKRQIGKYSIRDAS
LNSKLFSLAQGWGGKEEGLGLTSCAAENSCDQVSLQLGRTLHFEFYPSDE
SPSELPGGLQVIHVPDVSICAESDLELLNKLVIDHNVPPSLRFALLTRMR
FARAFSSLATRQQFTCIRLYAFVVLVQASGDTENVVSFFNGEPEFVNELV
TLVSYEDTVPEKIRILCLLSLVALSQDRTRQPTVLTAVTSGGHRGLLSGL
MQKAIDSVVCITSKWSLAFAEALLSLVTVLVSSSSGCSAMREAGLIPTLV
PLIKDTDPQHLHLVSAAVHILEAFMDYSNPAAALFRDLGGLDDTIFRLKL
EVSRTEDDVKEKNCSSDSNGPDTEQLPYSEALISYHRRLLLKALLRAISL
GTYAPGNTNLYGSEESLLPECLCIIFRRAKDFGGGVFSLAATVMSDLIHK
DPTCFNALDSAGLTSTFLDAISDEVICSAEAITCIPQCLDALCLNNSGLQ
AVKDRNALRCFVKIFTSPSYLRALTGDTPGSLSSGLDELLRHQSSLRTYG
VDMFIEILNSMLIIGSGMEATTSKSADVPTSAAPVPMEIDVDEKSLAVSD
EAEPSSDTSPANIELFLPDCVCNVARLFETVLQNAEVCSLFVEKKGIDAV
LQLFSLPLMPLSTSLGQSFSVAFKNFSPQHSAGLARIVCSYLREHLKKTK
ILLVSIEGTQLLKLESAIQTKILRSLSCLEGMLSLSNFLLKGSASVISEL
SAADADVLKELGITYKQTIWQMALCNDTKEDEKKSVDRGSDNSVSASSST
AERESDEDSSNALAVRYTNPVSIRSSSSQSIWGGDREFLSIVRSGEGIHG
RTRHAIARMRGGRTRRHLESFNFDSEIPADLPVTSSSHELKKKSTEVLIA
EILNKLNCTLRFFFTALVKGFTSANRRRIDGASLSSASKTLGTALAKVFL
EALNFDGYGAAAGHEKSLSVKCRYLGKVVDDITFLSFDTRRRVCFTAMVN
SFYVHGTFKELLTTFEATSQLLWTVPFSIPASSTENEKPGERNIWSHSKW

LVDTLQNYCRALDYFVNSTYLLSPTSQTQLLVQPASVGLSIGLFPVPREP
ETFVRNLQSQVLDVILPIWNHPMFPDCNPNFVASVTSLVTHIYSGVVDAR
ENRSGVTRGINQRALPLQLDESIVGMIVEMGFSRSRAEIALRRVGTNSVE
MAMDWLFTNPEQPVQEDDELAQALALSLGNSSETPKLEDTEKPVDVPQEE
AEPKEPPVDEVIAASQLKLVQLDFSKDTGALTMIPHILALVLSEDDNTRE
IAAQDGIVTVAIGILTDFNLKSESETEILAPKCISALLLVLSMMLQAQTK
LSSEYVEGNQGGSLVPSDSPQDSTAALKDALSSDVAKGESNQALELIFGK
STGYLTMEEGHKALLIACGLIKQHVPAMIMQAVLQLCARLTKSHALAIQF
LENGGLSSLFNLPKKCCFPGYDTVASVIVRHLVEDPQTLQIAMETEIRQT
LSGKRHIGRVLPRTFLTTMAPVISRDPVVFMKAVASTCQLESSGGRDFVI
LSKEKEKPKVSGSEHGFSLNEPLGISENKLHDVSGKCSKSHRRVPANFIQ
VIDQLIDLVLSFPRVKRQEDGETNLISMEVDEPTTKVKGKSKVGEPEKAS
SSRVGEPEKAEIPEKSEELARVTFILKLLSDIVLMYSHGTSVILRRDTEI
SQLRGSNLPDDSPGNGGLIYHVIHRLLPISLEKFVGPEEWKEKLSEKASW
FLVVLCSRSNEGRKRIINELSRVLSVFASLGRSSSKSVLLPDKRVLAFAN
LVYSILTKNSSSSSSNFPGCGCSPDVAKSMMDGGTIQCLTSILHVIDLDH
PDAPKLVTLILKSLETLTRAANAAEQLKSEVPNEKKNRDSDERHDSHGNS
TETEADELNQNNSSLQQVTDAAGNGQEQAQVSSQSAGERGSSQTQAMPQD
MRIEGDETILPEPIQMDFMREEIEGDQIEMSFHVENRADDDVDDDMGDEG
EDDEGDDEDADLVEDGAGVMSLAGTDVEDPEDTGLGDEYNDDMVDEDDDD
FHENRVIEVRWREALDGLDHFQILGRSGGGNGFIDDITAEPFEGVNVDDL
FALRRPLGFERRRQTGRSSLDRSGSEVHGFQHPLFSRPSQTGNTASVSAS
AGSISRHSEAGSYDVAQFYMFDTPVLPFDQVPVDPFSARLAGGGAPPPLT
DYSVVGMDSSRRGVGDSRWTDIGHPQPSSLSASIAQLIEEHFISNLRASA
PVNTVVERETNTTEIQEQLHPDVPPSVGSETVLGDGNEGGQQSEERELLN
NNENVNNPPDVMAESFAQGQANLASPVSQDTGESLQQLEVMQPLPLNSTP
NEIDRMEVGEGDGAPIDQVDHEAVHLISTAQGQPDTSSIQNVSVTAIAPP
VDDPDSNFQPSVDVDMSSDGAEGNQSVQPSPLDGDNNELSSMEATENVRN
DEQVEEGSLDGRAPEVNAIDPTFLEALPEDLRAEVLASQQAQSVQPPTYE
PPPVDDIDPEFLAALPPDIQTEVLAQQRAQRMVQQSQGQAVDMDNASIIA
TLPADLREEVLLTSSEAVLAALPSPLLAEAQMLRDRAMSHYQARSSVFGS
SHRLNNRRNGLGYNRLTGMDRGVGVTIGQRAVSSSADGLKVKEIEGDPLV
NADALKSLIRLLRLAQPLGKGLLQRLLLNLCAHSFTRANLVQLLLDMIRP
EMETSPSELAITNPQRLYGCQSNVVYGRSQLLNGLPPLVFRRVLEVLTYL
ATNHSAVADMLFYFDSSLLSQLSSRKGKEKVTHVTDSRDLEIPLVVFLKL
LNRPQLLQSTSHLGLVMGLLQVVVYTAASRIEGWSPSSGVPEKLENKPVG
EEASSETRKDAESELVGEADLSVARRKNCAEIYNIFLQLPQSDLCNLCIL
LGYEGLSDKIYSLAGEVLKKLAAVDVAHRKFFTKELSELASSLSSSTVRE
LATLSSKQKMSRSTGSMAGASILRVLQVLSSLTSPIDESNVGTERETEQE
EQNIMQRLNVALEPLWHELSQCISMTELQLDHTAAASNINPGDHVLGISP
TSSLSPGTQRLLPLIEAFFVLCEKIQTPSMLQQDTNVTAGEVKESSAHGS
SSKTSVDSQKKTDGSVTFSKFAEKHRRLLNSFIRQNPSLLEKSLSMMLKA
PRLIDFDNKKAYFRSRIRHQHDQHISGPLRISVRRAYVLEDSYNQLRMRS
PQDLKGRLNVQFQGEEGIDAGGLTREWYQLLSRVIFDKGALLFTTVGNDA
TFQPNPNSVYQTEHLSYFKFVGRMVAKALFDGQLLDVYFTRSFYKHILGV
KVTYHDIEAVDPDYYKNLKWLLENDVSDILDLTFSMDADEEKHILYEKTE
VTDYELKPGGRNIRVTEETKHEYVDLVAGHILTNAIRPQINAFLEGFNEL
IPRELVSIFNDKELELLISGLPEIDFDDLKANTEYTSYTAGSPVIHWFWE
VVKAFSKEDMARFLQFVTGTSKVPLEGFKALQGISGPQRLQIHKAYGAPE
RLPSAHTCFNQLDLPEYQSKEQLQERLLLAIHEASEGFGFA
>AT3G53090/UPL7
MDLNRKHKVSLRGASSGEISRDALLAKVSQERELRSYARRANAASLFIQR
VWRSYIVRKKAAIEIQEEWENLLSCHSVTLTKSWVSSRVLRPFLFFVRSL
SVQHQKIQAREIHCMQTCFKILLESINSNDQGYNFCSLAVGTSEDSKTWA
CQTRRMVSLCSFLLTECNYSQERIKDVIGVNALLLRILIVLTDPKSWKII
TNENFEDAETAKKIIIQFIGSCKSGYYSAVRRYIKTLTKHTDERLVITTS
AVTLALRPFHVKQPAFVDDNQPDTNLAVEEYVSLILTIPRLVCYLPSALI
RALKHKSILMPSFHTILLLKDKILNIISEMENSEKQSCTMEIPSVGWVIG
NIISLATVSETDFMDPQESNPEMFYVLYVHVIVTLAENLLSQVESVGIQD
IHLDIEATSNETEKGNSVKISFVEMLRPVCQQWHLAKLLAASGKEIRVIA
DKDASTSSKKGSETLGLLDIARLYSCMLRIFCVMNPVLGPLPVLNMLSFC
PGYIVSLWNSLESVLLPENGCTADDASHGSAKTSWNTRSPSEKKLKHLKN
DSVNKWVNVLNKFSGKSPGPREHVECTSDQPGSGQVNESTNDVWDVETLR
GGPVGISKEVSCLLHLFCATYAHLLVVLDDIQFYEKQVPFMLEKQQRIAS
MLNTLVYYGLLRGTGPESRQLMDSAIRCLHLLYERDCRHPFCASALWLSP
GRTSRPPIAFAARTHEVLPTSDVLTTPSMGSVITITPHVFPFEERVHVFR
EFISKDKASRKMAGEVDAPGARSIEIVVRRGHVVEDGFQQLNSIGSRLKS
SIHVSFVNESGLPEAGLDYGGLSKEFLTDITKAAFATEYGLFSQTPTSDR
LLVPSPSARHLENGIQMIEFLGRIVGKALYEGILLDYSFSHVFIQKLLGR
YSFIDELSGLDPELYRNLMYVKHYDGDLKELCLDFTVTEEFCGKMSIIEL
KPGGKDTSVTNENKMQYIHAMADYKLNRQIVPFSNAFYRGLTDLISPAWL
KLFNAHEFNQLLSGGNHDIDVDDLRRNTKYTGGYSDSSRTIKIFWEVMKG
FEPSERCLLLKFVTSCSRAPLLGFKYLQPTFIIHKVSCDTSLWAAIGGQD
VERLPSASTCYNTLKLPTYKRASTMREKLLYAITSNAGFELS
>AT1G70320/UPL2
MKLRRRRASEVPTKISLFINSVTSVPLELIQEPLASFRWEFDKGDFHHWV
DLFYHFDTFFEKHVKVRKDLRIEEEFDESDPPFPKDAVLQVLRVIRLVLE
NCTNKQFYTSYEQHLSLLLASTDADVVEACLQTLAAFLKRPTGKYSIRDA
SLNLKLFSLAQGWGGKEEGLGLTSCATEHSCDQLFLQLGCTLLFEFYASD
ESPSELPGGLQVIHVPDVSMRSESDLELLNKLVIDHNVPPSLRFALLTRL
RFARAFSSLATRQQYTCIRLYAFIVLVQASGDTENVVSFFNGEPEFVNEL

VTLVSYEDTVPAKIRILCLQSLVALSQDRTRQPTVLTAVTSGGHRGLLSG
LMQKAIDSVICNTSKWSLAFAEALLSLVTVLVSSSSGCSAMREAGLIPTL
VPLIKDTDPQHLHLVSTAVHILEVFMDYSNPAAALFRDLGGLDDTIFRLK
QEVSRTEDDVKEIVCCSGSNGPEDDTEQLPYSEALISYHRRLLLKALLRA
ISLGTYAPGNTNLYGSEESLLPECLCIIFRRAKDFGGGVFSLAATVMSDL
IHKDPTCFNALDSAGLTSAFLDAISDEVICSAEAITCIPQCLDALCLNNS
GLQAVKDRNALRCFVKIFSSPSYLKALTSDTPGSLSSGLDELLRHQSSLR
TYGVDMFIEILNSILIIGSGMEATTSKSADVPTDAAPVPMEIDVDEKSLA
VSDEAEPSSDTSPANIELFLPDCVCNVARLFETVLQNAEVCSLFVEKKGI
DTVLQLFSLPLMPLSTSLGQSFSVAFKNFSPQHSAGLARILCSYLREHLK
KTNNLLVSIEGTQLLKLESAVQTKILRSLSCLEGMLSLSNFLLKGSASVI
SELSAANADVLKELGITYKQTIWQMALCNDTKEDEKKSVDRASDNSVSAS
SSTAERESDEDSSNALAVRYTNPVSIRSSSSQSIWGGHREFLSVVRSGRG
VHGHTRHAIARMRGGRTRRHLESFNFDSEIPADLPVTSSSHELKKKSTEV
LIAEILNKLNCTLRFFFTSLVKGFTSANRRRIDGPSLSSASKTLGTALAK
VFLEALNFQGYGAAAGPDTSLSLKCRYLGKVVDDITFLTFDTRRRVCFTA
MVNSFYVHGTFKELLTTFEATSQLLWKVPFSIRASSTENEKSGERNLWSH
SKWLVDTLQNYCRALDYFVNSTYLLSPTSQTQLLVQPASVDLSIGLFPVP
REPETFVRNLQSQVLEVILPIWNHPMFPDCNPNFVASVTSLVTHIYSGVV
DTRENRSGATQGTNQRALPLQPDEAIVGMIVEMGFSRSRAEDALRRVGTN
SVEMAMDWLFTNPEDPVQEDDELAQALALSLGNSSETPKLEDTEKPVDVP
QEEAEPKEPPVDEVIAASVKLFQSDDSIAFPLVDLFVTLCNRNKGEDRPK
IVFYLIQQLKLVQLDFSKDTGALTMIPHILALVLSEDDNTREIAAQDGIV
AVAIGILTDFNLKSESETDILAPKCISALLLVLSMMLQAQTRLSSEYVEG
NQGGSLVLSDSPQDSTAALKDALSSDVAKGESNQALESMFGKSTGYLTME
ESSKVLLIACGLIKQRVPAMIMQAVLQLCARLTKSHALAIQFLENGGLSS
LFNLPKKCFFPGYDTVASVIVRHLVEDPQTLQIAMETEIRQTLSGKRHIG
RVLPRTFLTTMAPVISRDPVVFMKAVASTCQLESSGGTDFVILTKEKEKP
KVSGSEHGFSLNEPLGISENKLHDGSGKCSKSHRRVPTNFIQVIDQLIDI
VLSFPGLKRQEGEAANLISMDVDEPTTKVKGKSKVGEPEKAELGSEKSEE
LARVTFILKLLSDIVLMYLHGTSVILRRDTEISQLRGSNLPDDSPGNGGL
IYHVIHRLLPISLEKFVGPEEWKEKLSEKASWFLVVLCSRSNEGRKRIIN
ELTRVLSVFASLGRSSSQSVLLPDKRVLAFANLVYSILTKNSSSSNFPGC
GCSPDVAKSMIDGGTIQCLTSILNVIDLDHPDAPKLVTLILKSLETLTRA
ANAAEQLKSEVPNEQKNTDSDERHDSHGTSTSTEVDELNQNNSSLQQVTD
AVDNGQEQPQVSSQSEGERGSSLTQAMLQEMRIEGDETILPEPIQMDFFR
EEIEGDQIEMSFHVEDRADDDVDDDMDDEGEDDEGDDEDADSVEDGAGVM
SIAGTDVEDPEDTGLGDEYNDDMVDEDEEDEDEYNDDMVDEDEDDEDEYN
DDMVDEDEDDFHETRVIEVRWREALDGLDHFQIVGRSGGGNGFIDDITAE
PFEGVNVDDLFALRRSLGFERRRQTGRSSFDRSGSEVHGFQHPLFSRPSQ
TGNTASVSASAGSISRHSEAGSYDVAQFYMFDSPVLPFDQVPVDPFSDRL
GGGGAPPPLTDYSVVGMDSSRRGVGDSRWTDVGHPQPSSLSASIAQLIEE
HFITNLRASAPVDTVVERETNTTEVQEQQQPDVPPSVGSETVLGDGNEGG
EQSEEHELLNNNEVMHPLPLNSTPNEIDRMEVGEGGGAPIEQVDREAVHL
ISSAQGQSDTSGIQNVSVTAIPPPVDDPDSNFQPSVDVDMSSDGAEGNQS
VQPSPLDGDNNELSSMEATQDVRNDEQVDEGSLDGRAPEVNAIDPTFLEA
LPEDLRAEVLASQQAQSVQPPTYEPPSVDDIDPEFLAALPPEIQREVLAQ
QRAQRMLQQSQGQPVDMDNASIIATLPADLREEVLLTSSEAVLAALPPPL
LAEAQMLRDRAMRHYQARSRVFGSSHRLNNRRNGLGYRLTGMERGVGVTI
GQRDVSSSADGLKVKEIEGDPLVNADALKSLIRLLRLAQPLGKGLLQRLL
LNLCAHSFTRANLVQLLLDMIRPEMETLPSELALTNPQRLYGCQLNVVYG
RSQLLNGLPPLVFRRVLEVLTYLATNHSAVADMLFYFDSSLLSQLSSRKG
KEKVTHETDSRDLEIPLVVFLKLLNRPQLLQSTSHLALVMGLLQVVVYTA
ASRIEGWSPSSGVPEKLENKPVGEEASSETQKDAESELSVARRKNCAELY
NIFLQLPQSDLCNLCMLLGYEGLSDKIYSLAGEVLKKLAAVDVTHRKFFT
KELSELASGLSSSTVRVLATLSTTQKMSQNTCSMAGASILRVLQVLSSLT
STIDDSNVGTDKETDQEEQNIMQGLKVALEPLWQELGQCISMTELQLDHT
AATSNVNPGDHVLGISPTSSLSPGTQSLLPLIEAFFVLCEKIQTPSMLQQ
DATVTAGEVKESSTHGSSSKTIVDSQKKIDGSVTFSKFVEKHRRLLNSFV
RQNPSLLEKSFSMMLKAPRLIDFDNKKAYFRSRIRHQHDQHISGPLRISV
RRAYVLEDSYNQLRMRSPQDLKGRLNVQFQGEEGIDAGGLTREWYQLLSR
VIFDKGALLFTTVGNDATFQPNPNSVYQTEHLSYFKFVGRMVAKALFDGQ
LLDVYFTRSFYKHILGVKVTYHDIEAVDPDYYKNLKWLLENDVSDILDLT
FSMDADEEKHILYEKTEVTDYELKPGGRNIRVTEETKHEYVDLVADHILT
SAIRPQINAFLEGLNELIPRELVSIFNDKELELLISGLPEIDFDDLKANT
EYTSYTVGSPVIRWFWEVVKAFSKEDMARFLQFVTGTSKVPLEGFKALQG
ISGPQRLQIHKAYGSPERLPSAHTCFNQLDLPEYQSKEQVQERLLLAIHE
ANEGFGFA
